# The genetic architecture of a host shift: An adaptive walk protected an aphid and its endosymbiont from plant chemical defenses

**DOI:** 10.1126/sciadv.aba1070

**Published:** 2020-05-06

**Authors:** Kumar Saurabh Singh, Bartlomiej J. Troczka, Ana Duarte, Vasileia Balabanidou, Nasser Trissi, Leonela Z. Carabajal Paladino, Petr Nguyen, Christoph T. Zimmer, Kyriaki M. Papapostolou, Emma Randall, Bettina Lueke, Frantisek Marec, Emanuele Mazzoni, Martin S. Williamson, Alex Hayward, Ralf Nauen, John Vontas, Chris Bass

**Affiliations:** 1 College of Life and Environmental Sciences, Biosciences, University of Exeter, Penryn Campus, Penryn, Cornwall, UK.; 2 Institute of Molecular Biology & Biotechnology, Foundation for Research & Technology Hellas, Crete, Greece.; 3 Biology Centre of the Czech Academy of Sciences, Institute of Entomology, 370 05 České Budějovice, Czech Republic.; 4 University of South Bohemia, Faculty of Science, 370 05 České Budějovice, Czech Republic.; 5 Bayer AG, Crop Science Division, R&D, Alfred Nobel-Strasse 50, 40789 Monheim, Germany.; 6 Department of Sustainable Crop Production, Section Sustainable Crop and Food Protection, Università Cattolica del Sacro Cuore, Piacenza, Italy.; 7 Department of Biointeractions and Crop Protection, Rothamsted Research, Harpenden, UK.; 8 Department of Crop Science, Agricultural University of Athens, Athens, Greece.

## Abstract

Host shifts can lead to ecological speciation and the emergence of new pests and pathogens. However, the mutational events that facilitate the exploitation of novel hosts are poorly understood. Here, we characterize an adaptive walk underpinning the host shift of the aphid *Myzus persicae* to tobacco, including evolution of mechanisms that overcame tobacco chemical defenses. A series of mutational events added as many as 1.5 million nucleotides to the genome of the tobacco-adapted subspecies, *M. p. nicotianae*, and yielded profound increases in expression of an enzyme that efficiently detoxifies nicotine, both in aphid gut tissue and in the bacteriocytes housing the obligate aphid symbiont *Buchnera aphidicola*. This dual evolutionary solution overcame the challenge of preserving fitness of a mutualistic symbiosis during adaptation to a toxic novel host. Our results reveal the intricate processes by which genetic novelty can arise and drive the evolution of key innovations required for ecological adaptation.

## Teaser

A complex series of mutational events protected a mutualistic symbiosis during the shift of an insect to a toxic host plant.

## INTRODUCTION

The host shifts of phytophagous insects represent exceptional systems to study ecological adaptation and the process of speciation in action. The shift to a novel host and the evolution of adaptations that increase fitness on that host can lead to diversification and reproductive isolation and thus initiate speciation (*1*–*3*). Insect host shifts are also of great applied importance, as many insect species have become destructive agricultural pests by shifting from native hosts to crop plants (*4*). Despite the evolutionary and applied importance of this topic, the genetic events that allow herbivorous insects to “bridge the gap” and exploit a new host are poorly understood. In particular, current knowledge of the genetic architecture that underpins the key adaptations required to effectively utilize a novel host, and how this genetic variation is created in the first place, is extremely limited (*5*). Adding a further level of complexity, most plant sap–feeding insect species rely on obligate microbial symbionts that are essential for their survival. In these cases, whether the genomic mechanisms that facilitate insect host shifts are influenced by the additional requirement to preserve the fitness of these “hidden players” is unknown.

A prerequisite for the host shift of many phytophagous insects is the evolution of mechanisms to overcome the toxic secondary metabolites produced by the novel host plant as an antiherbivore defense. An excellent example of this phenomenon is the host shift of the aphid pest *Myzus persicae* to tobacco (*Nicotiana tabacum*). This plant-host shift required the evolution of a key adaptation: enhanced tolerance to nicotine, a potent natural insecticide produced by this plant (*6*). Tobacco-adapted races can be morphologically and genetically differentiated from *M. persicae* sensu stricto and have been characterized as a distinct subspecies, *M. persicae* ss*p. nicotianae* (*7*). Here, we exploited this system to gain new insights into the genetic bases of the key adaptations underpinning insect host shifts and the birth of new subspecies.

## RESULTS AND DISCUSSION

### Transcriptomic and genetic variation in an aphid subspecies

**Fig. 1:**
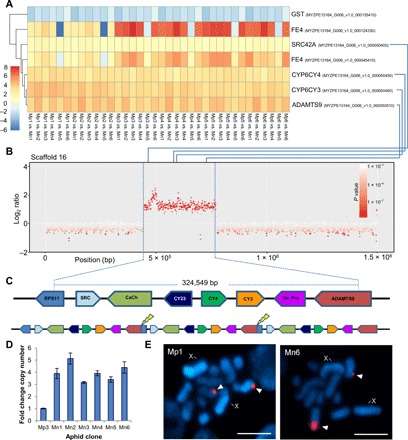
A large segmental duplication in *M. p. nicotianae* led to the amplification and overexpression of multiple genes. (**A**) Gene expression heat map showing genes consistently DE in 36 comparisons of *M. p. nicotianae* with *M. persicae s.s.* [6 *M. p. nicotianae* clones (Mn1 to Mn6) compared to 6 *M. persicae s.s.* clones (Mp1 to Mp6) are shown]; cell color indicates log_2_ fold change. Four of these genes localize to scaffold 16 [indicated by the blue lines linking (A) and (B)]. (**B**) Sliding window analysis of CNV between *M. p. nicotianae* and *M. persicae* across scaffold 16. In this representative plot, clone Mn3 was compared with clone Mp2; see data file S2 for the results of all 36 comparisons. (**C**) The region of elevated copy number includes some or all of the coding sequence of the genes, *RPS11*, *Src42A* (SRC), *T-type calcium channel* (CaCh), *CYP6CY23* (CY23), *CYP6CY4* (CY4), *CYP6CY3* (CY3), pseudogene of unknown function (Un_Pro) and *ADAMTS9*, and has been tandemly duplicated as a series of direct repeats. (**D**) Precise determination of copy number of the region amplified by qPCR in all *M. p. nicotianae* clones compared to *M. persicae* clone Mp3. Error bars indicate 95% confidence limits (*n* = 4). (**E**) Localization of *CYP6CY3* detected with tyramide-Cy3 (red, arrowheads) on metaphase chromosomes of Mp1 and Mn6 counterstained with 4′,6-diamidino-2-phenylindole (blue) by means of TSA-FISH. X, sex chromosome. Scale bar, 5 μm.

We first used a transcriptomic-led approach to examine whether a core suite of genes is consistently differentially expressed (DE) between *M. persicae* and *M. p. nicotianae*. Replicated transcriptomes of 12 aphid clones (6 of each subspecies) of diverse geographic origin were sequenced, and DE genes were called between each *M. p. nicotianae* clone and each *M. persicae* clone to yield a total of 36 comparisons (lists of DE genes) (data file S1). Comparison of these gene lists identified just seven genes that were consistently DE between all *M. persicae* and *M. p. nicotianae* clones (Fig. 1A). Of these, four were consistently up-regulated in *M. p. nicotianae* compared to *M. persicae*: the tyrosine-protein kinase *Src42A*, the cytochrome P450s *CYP6CY3* and *CYP6CY4*, and disintegrin and metalloproteinase with thrombospondin motifs 9 (*ADAMTS9*). A single gene was underexpressed in all 36 comparisons (a glutathione *S*-transferase belonging to the sigma class). All four overexpressed genes in the core gene set are located on scaffold 16 of the *M. persicae* genome, suggesting that they may be coregulated (Fig. 1B). As we have previously shown that *CYP6CY3* is amplified in *M. p. nicotianae* (*6*), we asked whether gene duplication/amplification also plays a role in the up-regulation of the other genes identified as consistently overexpressed in this subspecies. To answer this, we resequenced the genomes of the same 12 aphid clones used for RNA sequencing (RNA-seq) and looked for copy number variation (CNV) between the different *M. persicae* and *M. p. nicotianae* clones across scaffold 16. This analysis revealed significant (*P* < 0.0001 in all comparisons) and consistent increases in copy number in all *M. p. nicotianae* clones across a large region of scaffold 16 spanning a total of ~324,600 nucleotides (Fig. 1B, fig. S1A, and data file S2). The apparent copy number increase was further associated with genomic breakpoints at the boundaries of this region in the form of soft-clipped reads that diverge in sequence after crossing these positions and physically link these two loci (positions 16:445,716 and 16:770,265) (fig. S1B). This, together with the marked shift in coverage over this region, is an unmistakable signature of a direct tandem duplication of the entire region between the two breakpoints. Quantitative polymerase chain reaction (qPCR) analysis of copy number at six different positions across the amplicon indicated that this locus is three- to fivefold (average of fourfold) amplified in *M. p. nicotianae* clones (Fig. 1D). This equates to the addition of up to 1.5 million bases to the chromosome bearing these loci. Tyramide signal amplification fluorescence in situ hybridization (TSA-FISH) localized the segmental duplication to a single subtelomeric position on chromosome 3 (Fig. 1E and fig. S2), providing unequivocal evidence of amplification as a tandem array of repeats.

**Fig. 2:**
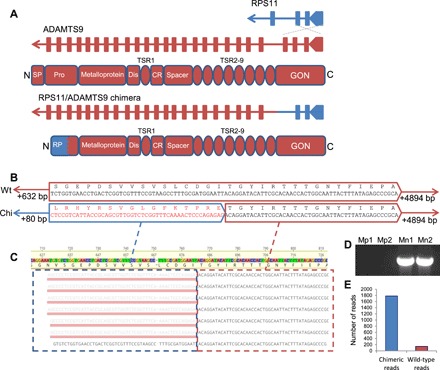
An *RPS11/ADAMTS9* chimeric gene is expressed in *M. p. nicotianae*. (**A**) The segmental duplication observed in *M. p. nicotianae* is predicted to create a chimeric gene fusing the promoter and first two exons of *RPS11* with the last 23 exons of *ADAMTS9*. This would result in the loss of the ADAMTS9 signal peptide (SP) and much of the prodomain (Pro). Disintegrin-like domain (Dis), cysteine-rich domain (CR), thrombospondin type 1 repeat (TSR). (**B**) De novo assembly of RNA-seq data from all *M. p. nicotianae* clones assembled a chimeric contig (bottom sequence) comprising a fusion of the *RPS11/ADAMTS9* gene that is not present in *M. persicae* (top sequence). Sequence from *RPS11* is boxed in blue and from *ADAMTS9* is boxed in red. Wt, wild-type. (**C**) Mapping *M. p. nicotianae* RNA-seq reads to the reference *ADAMTS9* gene reveals chimeric reads, and these represent >90% of the reads mapping to this region (**E**). (**D**) Reverse transcription PCR verification that the chimeric gene is expressed only in *M. p. nicotianae* as predicted.

The large genomic region amplified [324,549 base pairs (bp)] encompasses multiple genes (Fig. 1C). In addition to *Src42A*, *CYP6CY3*, *CYP6CY4*, and most of *ADAMTS9*, manual curation of this region of scaffold 16 revealed a gene encoding a voltage-dependent T-type calcium channel (along with additional upstream partial sequences with homology to the same protein), a third cytochrome P450 gene, *CYP6CY23*, that had been misannotated in the genome, and a predicted pseudogene of unknown function. Finally, the first two exons of the 40*S* ribosomal protein S11 (*RPS11*) gene and the last 23 exons of *ADAMTS9* are inside the 5′ and 3′ breakpoints of the segmental duplication, respectively (Fig. 2A). As the segmental duplication occurs as a direct tandem repeat, the chromosomal rearrangement is predicted to create a fusion of *RPS11* and *ADAMTS9* at the junctions between amplicon copies (Fig. 1C). Analysis of transcriptome assemblies of *M. p. nicotianae* clones and conventional PCR confirmed that the *RPS11/ADAMTS9* chimera is transcribed as predicted (Fig. 2, B to D).

### Gene amplification underpins key innovations

**Fig. 3:**
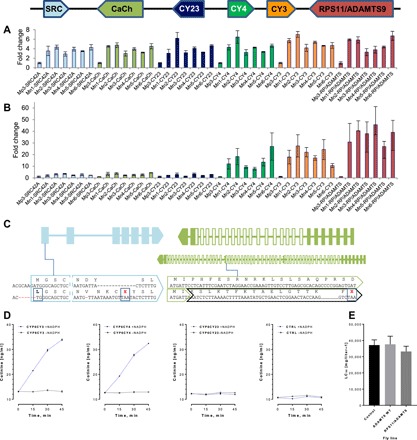
Molecular, bioinformatic, and functional characterization of candidate genes within the segmental duplication. (**A** and **B**) qPCR analysis of copy number (A) and mRNA expression (B) of genes within the segmental duplication. In each case, data are shown as fold change between the six *M. p. nicotianae* clones and *M. persicae* clone Mp3. Error bars indicate 95% confidence limits (*n* = 4). (**C**) Nonsense mutations are observed in exon 1 of certain copies of *Src42A* (light blue, left), and in the T-type calcium channel gene (green, right) caused by an internal duplication (duplicated region indicated by white boxes). In each case, the wild-type sequence is shown on top, and those carrying mutations are shown underneath. (**D**) Metabolism of nicotine by recombinant CYP6CY3, CYP6CY4, and CYP6CY23. Recovery of the nicotine metabolite cotinine over time in the presence (blue lines) or absence of NADPH (reduced form of nicotinamide adenine dinucleotide phosphate; black lines) is shown. Error bars display SD (*n* = 3). (**E**) Sensitivity of transgenic flies expressing *ADAMTS9* or *RPS11/ADAMTS9* to nicotine compared to a fly line of the same genetic background without a transgene (control). Sensitivity was measured by calculating lethal concentration 50 (LC_50_) values for each line. Error bars indicate 95% confidence limits (*n* = 5).

To summarize, a large chromosomal rearrangement in tobacco-adapted aphids has resulted in the amplification of a suite of genes and the creation of a new chimeric gene. But which of these genes provide a fitness benefit to *M. p. nicotianae* on tobacco at increased gene dosage? Changes in gene copy number and associated increases in gene dosage are usually detrimental (*8*); thus, nonfunctionalizing mutations accumulate very quickly in duplicate genes that confer no fitness advantage (*9*). We therefore predicted that genes in the segmental duplication that are neutral or have a fitness cost at increased gene dosage would accumulate mutations by drift, resulting in a reduction in their expression. In contrast, in the case of gene copies that provide a fitness benefit, deleterious mutations would be removed by purifying selection, and expression levels would remain high. We tested these predictions by interrogating DNA-sequencing (DNA-seq) data to screen for loss-of-function mutations in gene copies and alterations in the proximal promoter region of each gene and by comparing the copy number of each gene with the level of mRNA expression using qPCR (Fig. 3, A and B). A clear distinction was seen in the level of mRNA expression of genes within the segmental duplication with several genes expressed at lower levels than would be expected based on copy number alone. These included the tyrosine-protein kinase *Src42A*, the voltage-dependent T-type calcium channel, and the P450 *CYP6CY23* (average of 2.7-, 3.2-, and 2.4-fold mRNA overexpression, respectively) (Fig. 3, A and B). Examining DNA-seq reads mapped to these genes revealed evidence of genetic alterations that would affect the expression, transcript abundance, or translation of two of these genes. In the case of *Src42A*, in reads from all but one of the *M. p. nicotianae* clones, we observed a 6-bp deletion in certain gene copies (Fig. 3C) (~8 to 65% of the reads, depending on clone, table S1) that results in the removal of the first nucleotide encoding the methionine start codon, likely preventing or inhibiting translation of the mRNA produced. A 1-bp deletion and a 13-bp insertion in the same exon was also observed in certain *Src42A* gene copies (Fig. 3C) in four of the six *M. p. nicotianae* clones (15 to 34% of the reads, depending on clone, table S1). These alterations create a stop codon in exon 1 of the gene, and aberrant transcripts with this mutation are predicted to be removed by nonsense mediated decay. Mapping RNA-seq reads of *M. p. nicotianae* clones to this gene showed that this is indeed the case with altered transcripts detected but occurring at lower frequency (6 to 16%) than would be expected based on the frequency of this mutation in DNA-seq data from the same clones (table S1). An internal duplication was also observed in 24 to 48% of the reads mapping to the T-type calcium channel in all *M. p. nicotianae* clones, which results in the fusion of exon 24 with intron 7 creating nonsense mutations (Fig. 3C, fig. S3, and table S1). These findings clearly illustrate the imperfect process involved in adaption to a new host, with the initial amplification of a suite of genes followed by remodeling to eliminate gene copies that provide no fitness benefit, creating genetic by-product in the form of pseudogenes. Last, although no nonsense mutations were observed in copies of *CYP6CY23*, the reduced expression of this gene in comparison to its copy number (Fig. 3, A and B) does not support an adaptive role (see also below).

In contrast to the above genes, the P450s *CYP6CY4* and *CYP6CY3* and the *RPS11/ADAMTS9* fusion gene were expressed at higher levels than would be expected based purely on copy number (average of 14.7-, 19.9-, and 36.8-fold mRNA overexpression) (Fig. 3, A and B). Examination of the DNA-seq data of *M. p. nicotianae* clones mapped to these genes revealed no evidence of copies with loss-of-function mutations within coding sequences or polymorphisms within the putative promoter regions of different gene copies.

The substrate of ADAMTS9 in insects is unknown; however, in mammals, the orthologous gene processes the proteoglycans versican and aggrecan and thus plays a vital role in modulating the extracellular matrix (*10*). In the RPS11/ADAMTS9 chimera, the signal peptide and much of the prodomain (total of 227 amino acids) of ADAMTS9 have been lost (Fig. 2) and replaced with 43 amino acids of RPS11, while the catalytic and ancillary domains are unaffected. An additional consequence of the gene rearrangement is the replacement of the native *ADAMTS9* promoter with that of *RPS11* in the chimeric gene. Comparison of the expression level of wild-type *RPS11* and *ADAMTS9* in *M. persicae* reveals that the basal expression of *RPS11* is considerably higher than *ADAMTS9* (average fragments per kilobase of transcript per million mapped reads of 1086.32 versus 11.37). Thus, the high expression of the fusion gene in *M. p. nicotianae* (Figs. 2E and 3B) likely results from the promoter exchange. To examine whether the RPS11/ADAMTS9 chimera provides any fitness benefit in the presence of nicotine, we generated transgenic *Drosophila melanogaster* strains expressing either wild-type *ADAMTS9* or the chimeric *RPS11/ADAMTS9* and examined their sensitivity to nicotine. Flies expressing either transgene showed no increase in tolerance to nicotine compared to flies of the same genetic background lacking a transgene (Fig. 3E). These findings suggest that the chimeric gene provides no protection to *M. p. nicotianae* from nicotine, and either provides some other fitness benefit on tobacco or is a neutral polymorphism that has been retained because of its close linkage to other genes in the amplicon that are under positive selection (see below).

The three P450 genes observed in the amplicon, *CYP6CY3*, *CYP6CY4*, and *CYP6CY23*, represent historical duplicates and share 72 to 81% amino acid sequence identity. We have previously shown that CYP6CY3 provides a fitness benefit to *M. p. nicotianae* by efficiently detoxifying nicotine (*6*). To examine whether the two other P450s perform the same function, we individually expressed each of the three P450s in combination with cytochrome P450 reductase (CPR) from *D. melanogaster*, in an insect cell line. Incubation of the microsomal preparations obtained with nicotine demonstrated that both CYP6CY4 and CYP6CY3 are highly effective at metabolizing nicotine to its nontoxic metabolite cotinine (Fig. 3D). In contrast, CYP6CY23 showed no capacity to metabolize nicotine in vitro (Fig. 3D). Thus, the overexpression of *CYP6CY3* and *CYP6CY4*, but not *CYP6CY23*, allowed *M. p. nicotianae* to overcome the primary defensive allelochemical produced by tobacco plants.

### Spatial expression of CYP6CY3 is key to fitness

Although CYP6CY3 and CYP6CY4 process nicotine with similar efficiency and show parity in their level of expression in whole adult *M. persicae*, our RNA-seq data reveal that, in *M. p. nicotianae*, the expression of *CYP6CY3* is more than double that of *CYP6CY4* (fig. S4A). This finding was unexpected given the equivalent copy number of these two P450s in the segmental duplication and suggests that CYP6CY3 may confer greater fitness benefits to the tobacco-adapted subspecies.

**Fig. 4:**
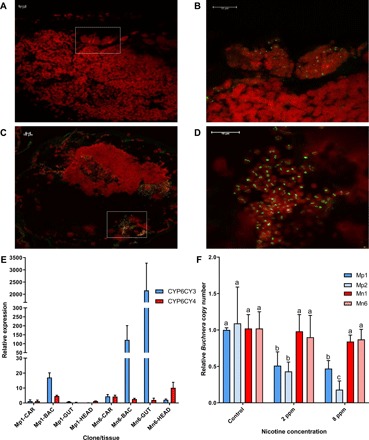
Overexpression of CYP6CY3 in the bacteriocyte and the gut of *M. p. nicotianae* protects this aphid subspecies and its obligate endosymbiont *Buchnera aphidicola* from nicotine. (**A** to **D**) Immunohistochemical localization of CYP6CY3 (green signal) in the bacteriocytes of *M. persicae* [(A) and (B)] and *M. p. nicotianae* (C) and (D). Nucleic acids stained with To-PRO 3-Iodide (red signal). (**E**) Expression of *CYP6CY3* and *CYP6CY4* in the carcass (CAR), gut, bacteriocyte (BAC), and head of the *M. persicae* clone Mp1 and *M. p. nicotianae* clone Mn6 as determined by qPCR. Error bars indicate 95% confidence limits (*n* = 4). (**F**) Titer of *B. aphidicola* in two clones of *M. persicae* (blue bars) and two clones of *M. p. nicotianae* (red bars) after feeding on a diet with [2 or 8 parts per million (ppm)] or without nicotine (control). Error bars indicate 95% confidence limits (*n* = 4). Significant differences (*P* < 0.05) in expression between the different treatments and the control are denoted using letters above bars as determined by one-way analysis of variance (ANOVA) with post hoc Tukey honestly significant difference.

**Fig. 5:**
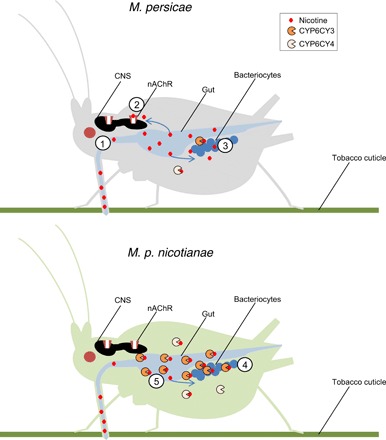
Schematic of the evolution of the molecular innovations in *M. p. nicotianae* that provide protection from toxic nicotine. Nicotine (1) is taken up via the gut during feeding and causes toxicity to *M. persicae* via its action at the nicotinic acetylcholine receptor (nAChR) (2) and on the obligate endosymbiont *B. aphidicola* in aphid bacteriocytes (3). In *M. p. nicotianae*, chromosomal rearrangements result in the increased expression of CYP6CY3 and CYP6CY4, which detoxify nicotine. In the case of CYP6CY3, expression is significantly enhanced in bacteriocytes (4) and reprogrammed to include the aphid gut (5) providing two lines of defense that protect *M. p. nicotianae* and its symbiont from this secondary metabolite. CNS, central nervous system.

To gain further insights into its function, we raised specific antibodies against CYP6CY3 and examined where the enzyme is expressed using immunohistochemistry. Unexpectedly, immunolocalization of CYP6CY3 revealed that the primary site of expression of this P450 in *M. persicae s.l.* are the bacteriocytes, specialized aphid cells that house the obligate endosymbiont *Buchnera aphidicola*, which provides essential amino acids and other nutrients to its host (Fig. 4, A to D) (*11*). A stronger signal was observed in the bacteriocytes of *M. p. nicotianae* than *M. persicae*, consistent with the overexpression of this P450 in the tobacco-adapted subspecies. To verify this unexpected finding, we used qPCR to examine the expression of *CYP6CY3* and *CYP6CY4* in dissected bacteriocytes, guts, and heads of *M. p. nicotianae* and *M. persicae* clones used in this study (Fig. 4E). We further exploited publicly available RNA-seq data to examine the expression of these P450s in whole insects, guts, and bacteriocytes of *M. p. nicotianae* and *M. persicae* clones collected in the United States (fig. S4B). These analyses confirmed the clear tissue-specific expression of *CYP6CY3* in bacteriocytes in both subspecies, with a sevenfold greater expression observed in bacteriocytes of *M. p. nicotianae* compared to *M. persicae* (Fig. 4E and fig. S4B). Unexpectedly, however, qPCR and RNA-seq analyses also revealed a second site of high expression exclusively in *M. p. nicotianae*, with expression of *CYP6CY3* in the gut of this subspecies >2500-fold higher than in guts of *M. persicae* (Fig. 4E and fig. S4B). While attempts to immunolocalize CYP6CY3 in aphid guts were unsuccessful, Western blotting confirmed the overexpression of the CYP6CY3 protein in the microsomal fraction of guts of *M. p. nicotianae* (fig. S4, D and E). In contrast to the findings on *CYP6CY3*, no clear pattern of tissue-specific expression of *CYP6CY4* was observed (Fig. 4E and fig. S4B). Thus, these data reveal a previously undescribed tissue-specific expression pattern for an insect P450 (the aphid bacteriocyte) while also uncovering profound modifications in the tissue-specific expression of CYP6CY3 in the tobacco-adapted subspecies. The native role(s) of CYP6CY3 in the aphid bacteriocyte is unclear. However, in relation to the host shift of *M. persicae* to tobacco, it is notable that nicotine has strong antimicrobial properties extending to Gram-negative bacteria such as *Escherichia coli* (*12*, *13**)*, which is the closest free-living relative of *B. aphidicola* (*11*). This led to a further question: Does the up-regulation of CYP6CY3 in *M. p. nicotianae* protect *B. aphidicola* from the inhibitory effects of nicotine? To investigate this, we fed adult *M. persicae* and *M. p. nicotianae* on an artificial diet containing a sublethal concentration of nicotine for 4 days and then determined the titer of *B. aphidicola* in aphids at the end of the experiment. A significant reduction in bacterial titer was observed in *M. persicae* clones fed a diet containing nicotine compared to their clone mates fed on a diet free of this compound (Fig. 4F). In contrast, no significant reduction in *B. aphidicola* titer was observed in *M. p. nicotianae* fed on the same concentrations of nicotine (Fig. 4F). These findings provide clear evidence that CYP6CY3 protects *B. aphidicola* from nicotine in the tobacco-adapted subspecies. While nicotine has antimicrobial properties, its toxicity to insects primarily results from its action on the nervous system where it disrupts nerve signaling by binding to nicotinic acetylcholine receptors, pentameric ligand-gated ion channels essential for fast synaptic transmission. Thus, expression of CYP6CY3 in the bacteriocyte alone would be insufficient to protect the aphid host from the primary mode of action of this toxin. The remodulation of the expression of CYP6CY3 in this subspecies to include the gut would therefore provide a first line of defense against the ingestion of nicotine during feeding. Together, these findings reveal an innovative dual evolutionary solution to overcome the primary antiherbivore defense of tobacco, allowing *M. persicae* to effectively exploit this plant (illustrated in Fig. 5).

### Complex mutational events at the CYP6CY3 locus

**Fig. 6:**
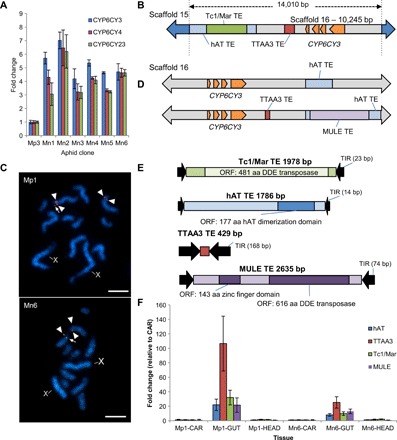
Further amplification of *CYP6CY3* is associated with the insertion of transposable elements that are highly expressed in the aphid gut. (**A**) Copy number of *CYP6CY3*, *CYP6CY4*, and *CYP6CY23* in *M. p. nicotianae* clones Mn1–6 compared to *M. persicae* clone Mp3 as determined by qPCR. Error bars indicate 95% confidence intervals (*n* = 4). (**B**) Schematic of the *CYP6CY3* amplicon obtained from BAC sequencing. The insertion sites of a Tc1/Mariner (Tc1/Mar), hAT, and TTAA3 transposable element are illustrated. (**C**) Colocalization of the BAC clone bearing a copy of *CYP6CY3* on scaffold 15, with scaffold 16 on metaphase chromosomes of Mp1 and Mn6. The scaffold 16 probe was detected with tyramide–fluorescein isothiocyanate (green, arrows), and the BAC was directly labeled with Cy3 (red, arrowheads). Note that only one scaffold 16 locus was detected; this was due to a limitation of TSA-FISH to provide balanced signals, rather than its absence in the homologous chromosome. X, sex chromosome. Scale bar, 5 μm. (**D**) Additional long single-molecule sequencing identified copies of *CYP6CY3* at the scaffold 16 locus in combination with the hAT element or in combination with the TTAA3, HAT, and Mutator-like (MULE) elements. (**E**) Features of the four transposons found in association with *CYP6CY3*. ORF, open reading frame; aa, amino acid. (**F**) Expression of hAT, TTAA3, Tc1/Mariner, and MULE in carcass, gut, or head tissue of *M. persicae* clone Mp1 and *M. p. nicotianae* clone Mn6. Error bars indicate 95% confidence limits (*n* = 4).

We hypothesized that the quantitative and qualitative changes in the expression of *CYP6CY3* observed in *M. p. nicotianae* were caused by additional mutational events that occurred before or after the large segmental duplication. To explore this, we examined DNA-seq reads mapped to the genomic regions flanking *CYP6CY3* and observed evidence of an additional genomic breakpoint in all *M. p. nicotianae* clones in 5 to 12% of sequence reads 3428 bp upstream of the gene. The divergent region of reads crossing this breakpoint all match a position on an alternative scaffold (scaffold 15) (fig. S5A), suggesting that *M. p. nicotianae* may have an additional copy of *CYP6CY3* at a novel locus. This was supported by qPCR analysis of gene copy number, which confirmed that most *M. p. nicotianae* clones have a higher copy number of *CYP6CY3* than the two other P450 genes present in the segmental duplication (10 copies compared to 8 copies of *CYP6CY4* and *CYP6CY23*) (Fig. 6A). To investigate this further, we screened a bacterial artificial chromosome (BAC) library that we created for one of the *M. p. nicotianae* clones (Mn4) using primers flanking this breakpoint. A positive clone was identified, which, when sequenced, revealed a single copy of the *CYP6CY3* gene flanked by sequence from scaffold 15. This confirms that an additional copy of *CYP6CY3* has inserted into a region of the genome distinct from the position of the segmental duplication (Fig. 6B and fig. S5, B and C). The additional *CYP6CY3* gene copy is part of an amplicon of 14,010 bp in size, including >3 kb of the *CYP6CY3* promoter, and has inserted into an intron of the ecdysone-induced 78C gene (fig. S5B). FISH using the BAC sequence as a probe localized the additional copy of *CYP6CY3* to a subtelomeric region on the same chromosome as the segmental duplication (Fig. 6C). Unexpectedly, examination of the BAC sequence revealed two transposon-like sequences downstream of *CYP6CY3* and adjacent to the 5′ break point, neither of which are observed on scaffold 15 or 16 in the reference *M. persicae* genome at this position (Fig. 6B and fig. S5C). The first of these is a nonautonomous DNA transposable element bearing the hallmarks of the hAT (hobo-Ac-Tam3) superfamily [8-bp target site duplication (TSD) and conserved C-terminal dimerization domain] (Fig. 6E). A second DNA transposon, which is apparently autonomous and shares closest sequence similarity to the Tc1/mariner superfamily, has inserted into the hAT element (Fig. 6E and fig. S5C). These nested insertions occur immediately adjacent to the 5′ breakpoint, providing clear evidence that they played a role in the mobilization of *CYP6CY3* to the new loci, either indirectly by acting as substrates for nonallelic homologous recombination or directly via alternative transposition. A third smaller element (429 bp) was also observed in the BAC-derived sequence, internal to the breakpoint, and 1540 bp downstream of *CYP6CY3* that had high sequence similarity to the TTAA3_AP element of *Acyrthosiphon pisum* (Fig. 6, B and E). To investigate whether any of these transposable element (TE) insertions are present adjacent to the other *CYP6CY3* copies in *M. p. nicotianae*, we sequenced the *M. p. nicotianae* clone Mn6 using single-molecule sequencing and mapped the long reads obtained to the *CYP6CY3* locus on scaffold 16 and the BAC sequence. In combination with additional mapping of the Illumina sequence reads obtained from *M. p. nicotianae* and *M. persicae* clones, this revealed a remarkable series of transposable element insertions downstream of *CYP6CY3* in *M. p. nicotianae* (Fig. 6, B and D and fig. S6). These include all the elements identified in the initial BAC sequence, together with a fourth TE belonging to the Mutator-like element (MULE) superfamily of DNA transposons (Fig. 6E). These elements occur individually or in combination, and we were able to make use of this to order the sequential mutational events at the *CYP6CY3* locus and draw the following conclusions: (i) All *M. persicae* clones lack the TE insertions on scaffold 16 or 15; (ii) all gene copies of *CYP6CY3* in *M. p. nicotianae* are associated with the hAT element, thus its insertion predates the segmental duplication; (iii) some but not all copies of *CYP6CY3* linked to their native position on scaffold 16 have the TTAA_3 and MULE insertions, thus these inserted after the segmental duplication, and in the case of TTAA_3 before the copying of *CYP6CY3* to scaffold 15; and (iv) the Tc1/mariner element could not be linked to a copy of *CYP6CY3* associated with scaffold 16, thus it occurred after the copying of *CYP6CY3* to scaffold 15. A schematic of the sequential steps in the amplification of *CYP6CY3* is shown in fig. S7. This analysis reveals that the copying of *CYP6CY3* to scaffold 15 occurred after the segmental duplication. Thus, this finding is consistent with theoretical predictions that mutations of large effect drive initial adaptive steps, while subsequent mutations with smaller fitness effects provide fine-tuning of novel functions (*14*).

The accumulation of TE insertions downstream of *CYP6CY3* is unprecedented in the segmental duplication. TEs are frequently enriched in promoters and other regions of genes and can drive both quantitative and qualitative changes in the expression of adjacent genes (*15*, *16*). When we examined the spatial expression of the four elements in *M. persicae* and *M. p. nicotianae*, we found that, in both subspecies, all four TEs are highly and specifically expressed in the gut (Fig. 6F), indicating that they carry gut-specific cis-regulatory elements. Thus, in addition to facilitating quantitative changes in *CYP6CY3* expression in *M. p. nicotianae* (by mediating further increases in the copy number of this P450), TE insertions may have played a role in the qualitative changes in its expression by bringing tissue-specific enhancer sequences into close proximity with this P450.

## CONCLUSION

Collectively, our findings provide fundamental insights into the evolutionary processes underlying the initial genotypic and phenotypic changes that facilitate insect adaptation to a novel ecological niche, in this case, exploitation of a novel host plant. The mutational source of the genetic and biological diversity observed in different insect host races or subspecies is a long-standing question in the field (*2*, *17*). Many studies on this topic have focused on the role of point mutations (single-nucleotide polymorphisms) in the evolution of key innovations required for host adaptation (*2*). Our findings demonstrate that more profound genetic alterations, involving chromosomal rearrangements and transposable element insertions, can be equally, or potentially even more, important in providing the initial raw material for adaptation and may provide greater opportunities for saltatory evolution. We demonstrate that such mutational events can add substantial new content (i.e., millions of nucleotides) to the genomes of insect host races. How many mutational events or “adaptive steps” are required to produce a new function is a central question in evolutionary biology. Our study illustrates how the evolution of even a relatively simple novel phenotype, resistance to nicotine, can be underpinned by a remarkable continuum of both large and small mutational events. These include whole-scale amplification of a suite of genes, nonfunctionalizing mutations that inactivate gene copies that provide no fitness benefit, transposable element insertions leading to further increases in the copy number of genes that are adaptive, and fundamental changes in gene regulation. The complexity that we observe in this case likely relates, at least in part, to the evolutionary challenge represented by the intimate relationship between plant sap–feeding insect species and their obligate endosymbionts. Our findings demonstrate how preserving the fitness of both members of these mutualistic symbioses during adaptation to a new ecological niche can be key to ecological and evolutionary success.

## MATERIALS AND METHODS

### Aphid clones

The *M. p. nicotianae* clones used in this study were collected from tobacco in Italy (Mn1 and Mn2), Greece (Mn3 and Mn4), and Zimbabwe (Mn5 and Mn6). The *M. persicae* clones were collected from Italy (Mp1 and Mp2) from peach and potato, respectively, from the UK (Mp3, Mp5, and Mp6) from potato, sugar beet, and oil seed rape, respectively, and from Germany (Mp4) from an unknown weedy host plant. All clones were reared asexually on individual Chinese cabbage leaves (*Brassica napus* L var. *chinensis* cv Tip-Top) in small plastic cups maintained at 18°C under a 16:8-hour light:dark regime for at least 1 year before molecular analyses. For cytogenetic studies, clones Mp1 and Mn6 were reared on pea as described previously (*18*).

### Nicotine bioassays and calculation of *B. aphidicola* copy number

Nicotine (Sigma) was directly dissolved into an aphid artificial diet (*19*) and filter-sterilized. A total of 0.5 ml was pipetted onto the end of a plastic cylinder (size: 25 mm deep, 25-mm internal diameter) that had been sealed with stretched Parafilm. A second layer of Parafilm was then stretched on top of this to form a sachet. Ten age-synchronized adult apterous aphids were transferred into the cylinder and the remaining opening sealed with Parafilm. Aphids were kept for 96 hours at 20°C under a 16:8-hour light:dark regime and then snap-frozen in liquid nitrogen for molecular analyses. Four replicates were used for each concentration. Control aphids were fed on artificial diet without nicotine under identical conditions. DNA was extracted from each pool of 10 aphids using the E.Z.N.A. Insect DNA Kit (Omega Bio-tek) and the titer of *B. aphidicola* measured by comparing the copy number of the *Buchnera*-specific gene *GroEL* with the *M. persicae*–specific genes actin and *para* (encoding the voltage-gated sodium channel) in qPCR. Primers are detailed in table S2 and qPCR methods were as detailed below.

### RNA-seq and identification of DE genes

Aphid clones were age-synchronized under identical environmental conditions, and four biological replicates each comprising 10 apterous female aphids 14 days in age were pooled for RNA extraction using the ISOLATE II RNA Mini Kit (Bioline). RNA was used as a template for the generation of barcoded libraries (TrueSeq RNA library preparation, Illumina), which were sequenced by the Earlham Institute (Norwich, UK), with the 48 samples multiplexed for sequencing across six lanes of an Illumina HiSeq 2500 using 100-bp paired-end sequencing. FastQC was used to check the quality of the raw reads obtained (*20*) and reads trimmed using Trim Galore (*21*). The Tuxedo workflow was used to map the reads of each clone against the annotated reference genome using TopHat, to estimate gene expression with Cufflinks and test for differential expression between each *M. persicae* clones and each *M. p. nicotianae* clone with Cuffdiff (*22*).

### DNA-seq, assembly, and CNV

Genomic DNA was extracted from pools of 10 aphids as above and used to construct PCR-free libraries. Libraries of the Mp3 and Mn4 clones were sequenced across a single lane of an Illumina HiSeq 2500 using a 100-bp paired-end read metric. The remaining libraries of the 10 other clones were multiplexed and sequenced across five lanes of an Illumina HiSeq 2500 with a 250-bp paired-end read metric. Coverage obtained ranged from 38 to >200×. The quality of the DNA-seq reads was checked and trimmed as above. The reads of each clone were individually mapped to scaffolds of interest of the reference *M. persicae* genome (clone G006) using Burrows-Wheeler Aligner (BWA) (*23*) or the map to reference function of Geneious version R9. To estimate gene copy number, the reads of each clone were mapped to the reference genome, and CNV was estimated using CNVseq (*24*) with data of each *M. persicae* clone compared to each *M. p. nicotianae* clone.

### Quantitative polymerase chain reaction

qPCR was used to examine the expression of genes of interest and changes in gene copy number as described previously (*6*) using the primers shown in table S2. Data were analyzed according to the ΔΔC_T_ method (*25*), using the geometric mean of two housekeeping genes (actin and *para*) for normalization according to the strategy described previously (*26*).

### Transgenic expression of candidate genes in *D. melanogaster*

Candidate genes of interest were synthesized (GeneArt, CA, USA) and subcloned into pUASattB40. Using the PhiC31 system, clones were transformed into the germ line of a *D. melanogaster* strain carrying the attP40 docking site on chromosome 2 [“y^1^w^67c23^; P attP40”, “1;2”]. The transgenic lines obtained were balanced, and the integration of genes were confirmed by PCR and sequencing using primers detailed in table S2. Virgin females of the Act5C-GAL4 strain were crossed with UAS-gene-of-interest males. Bioassays were used to assess the susceptibility of adult female flies to nicotine. Several concentrations of each compound were overlaid onto fly food in standard vials and allowed to dry for 4 hours. Fifteen to 20 adult flies (2 to 5 days post-eclosion) were then added to each vial, and the mortality by oral ingestion was assessed after 48 hours. Mortality was compared with control progeny (flies of the same genetic background but without transgenes). Five replicates were carried out for each concentration. Control mortality was assessed using tubes containing food minus insecticide. The relationship between concentration and mortality was determined using probit analysis with lethal concentration 50 (LC_50_) values and their respective 95% confidence interval values calculated in LeOra Software PoloPlus version 1.0.

### Heterologous expression of P450s

Candidate P450s and the housefly (*Musca domestica*) NADPH (reduced form of nicotinamide adenine dinucleotide phosphate)–dependent CPR (GenBank accession Q07994) were codon-optimized for expression in Sf9 cells, obtained by gene synthesis (Geneart), and inserted into the pDEST8 expression vector (Invitrogen). The PFastbac1 vector with no inserted DNA was used to produce a control virus. The recombinant baculovirus DNA was constructed and transfected into Sf9 insect cells (Gibco) using the Bac-to-Bac baculovirus expression system (Invitrogen) according to the manufacturer’s instructions. The titer of the recombinant virus was determined following standard protocols of the supplier. Sf9 cells were maintained in suspension culture under serum-free conditions (SF-900 II SFM, Gibco) at 27°C containing gentamycin (25 μg/ml; Gibco). Insect cells grown to a density of 2 × 10^6^ cells/ml were coinfected with recombinant baculoviruses containing P450 and CPR at various MOI (multiplicity of infection) ratios to identify the best conditions. Control cells were coinfected with the baculovirus-containing vector with no insert (ctrl-virus) and the recombinant baculovirus expressing CPR using the same MOI ratios. Ferric citrate and δ-aminolevulinic acid hydrochloride were added to a final concentration of 0.1 mM at the time of infection and 24 hours after infection to compensate the low levels of endogenous heme in the insect cells. After 60 hours, cells were harvested, washed with phosphate-buffered saline (PBS), and microsomes of the membrane fraction prepared according to standard procedures and stored at −80°C (*27*). P450 expression and functionality were estimated by measuring CO-difference spectra in reduced samples (*27*). The protein content of samples was determined using Bradford reagent (Sigma) and bovine serum albumin as a reference.

### Metabolism assays and ultra-performance liquid chromatography tandem mass spectrometry analysis

Metabolism of nicotine was assayed by incubating recombinant P450/CPR (2 pmol P450 per assay) or ctrl-virus/CPR microsomes in 0.1 M potassium phosphate buffer with an NADPH-regenerating system [Promega; 1.3 mM NADP^+^, 3.3 mM glucose-6-phosphate, 3.3 mM MgCl_2_, and glucose-6-phosphate dehydrogenase (0.4 U/ml^−1^)] and substrate (12.5 μM) at 27°C for 1 hour. The total assay volume was 200 μl using three replicates for each data point. Microsomes incubated without NADPH served as a control. The assay was quenched by the addition of ice-cold acetonitrile (to 80% final concentration), centrifuged for 10 min at 3000*g*, and the supernatant subsequently was analyzed by tandem mass spectrometry as described previously (*28*). For the chromatography, acetonitrile/water/0.1% formic acid was used as eluent in gradient mode. For detection and quantification, the multiple reaction monitoring (MRM) transitions 163 > 117 (nicotine); 177 > 80 (cotinine) were monitored. Recovery rates of parent compounds using microsomal fractions without NADPH were normally close to 100%. Substrate turnover from two independent reactions were plotted versus controls using GraphPad Software version 7.

### Creation of a BAC library and screening for *CYP6CY3*

Approximately 10 g of all developmental stages (nymphs and winged and nonwinged adults) of the *M. p. nicotianae* clone Mn4 were used to create a 10× pooled Hind III BAC library by Bio S&T (Quebec, Canada). High–molecular weight DNA was cloned into the plndigoBAC–Hind III vector and propagated in DH10B *E. coli* cells. A single positive clone carrying one copy of *CYP6CY3* in association with scaffold 15 of the *M. persicae* genome was identified by Bio S&T using colony PCR and the primers detailed in table S2. This was then propagated in 250 ml of LB medium containing chloramphenicol (12.5 mg/ml) and high-quality BAC DNA isolated using the Large-Construct Kit (Qiagen) following the manufacturer’s protocol. The bacmid construct was sequenced using PacBio sequencing on a single single-molecule real-time sequencing (SMRT) cell using C4 P6 chemistry by the Earlham Institute. Raw reads were assembled using Hierarchical Genome Assembly Process (HGAP) version 3 (*29*), and the assembly was manually annotated using Geneious software version 8.01 (Biomatters).

### Cytogenetic analysis

Spread chromosome preparations from clones Mp1 and Mn6 were obtained from embryos following the methods of Traut (*30*). The preparations were dehydrated in an ethanol series (70, 80, and 100%, 30 s each) and stored at −20°C until further use. Fragments of *CYP6CY3* and scaffolds 15 and 16 were amplified from genomic DNA of clone Mp3 by PCR (conditions are detailed in table S3 and primers in table S2) and cloned into pGEM-T Easy Vector (Promega). For BAC-FISH experiments, the BAC containing the *CYP6CY3* gene copy in the scaffold 15 locus was extracted using the Qiagen Plasmid Midi Kit (Qiagen). Simple and double TSA-FISH experiments were performed as described previously (*31*), but with the slight modifications described in table S3. In the case of BAC-FISH combined with TSA-FISH, either BAC-FISH was followed by TSA-FISH or vice versa. When BAC-FISH was followed by TSA-FISH, the slides were pretreated following the TSA-FISH protocol with modifications (see table S3), and BAC-FISH was then performed using the protocol described previously (*32*). The preparations were then stripped for reprobing, covered with probe cocktail prepared according to the TSA-FISH protocol, and processed accordingly. When TSA-FISH was followed by BAC-FISH, after tyramide incubation, the slides were denatured and the BAC probe was added to the slides. Results from all experiments were documented using a Zeiss Axioplan 2 microscope (Carl Zeiss) equipped with appropriate fluorescence filter sets and a cooled charge-coupled device camera using AnalySIS software, version 3.2 (Soft Imaging System GmbH). The images were pseudocolored and superimposed with Adobe Photoshop CS6 (Adobe Systems). To identify the chromosomes bearing *CYP6CY3*, 25 mitotic metaphases of Mp1, where *CYP6CY3* was mapped in both homologs using simple TSA-FISH, was used. The length of all the chromosomes was measured using MicroMeasure version 3.3 (*33*), and the percentage of the total complement length of chromosomes bearing *CYP6CY3* was calculated. We compared the obtained value (8.64% ± 0.001 SE) with those described previously (*18*) for each of the six pairs of chromosomes of the same clone.

### Immunohistochemistry and Western blots

Rabbit polyclonal antibodies targeting two specific CYP6CY3 peptides, corresponding to the amino acids 166 to 178 (peptide I: TNIDKCLRGGNE) and 410 to 425 (peptide II: IYSLHFDDKYFEDPQ), were produced by Davids Biotechnologie. Following a standard immunization protocol using a single rabbit, two peptide antibodies (anti-TNI and anti-IYS) were affinity-purified from rabbit serum using two affinity columns, with the corresponding peptides immobilized (peptide I and II, respectively). Peptide antibody reactivity against recombinant CYP6CY3 protein from bacteria was validated by Western blot (fig. S4C), and only the anti-IYS antibody was used for immunohistochemical experiments.

Five- to 7-day-old aphids were fixed in a cold solution of 4% formaldehyde (methanol free; Thermo Fisher Scientific) in PBS for 3 hours, cryoprotected in 30% (w/v) sucrose/PBS at 4°C for 12 hours, immobilized in optimal cutting temperature compound (Tissue-Tek; Sakura), and stored at −80°C until use. Immunofluorescence analysis, followed by confocal microscopy, was performed on longitudinal sections of the frozen prefixed aphid specimens. Briefly, 6-μm sections, obtained in cryostat with Ultraviolet C (UVC) disinfection (Leica CM1850UV), were washed (3 × 5 min) with 0.2% Tween in PBS, followed by a 10-min wash with 0.2% Triton/PBS, washed again (3 × 5 min) with 0.2% Tween/PBS, and blocked for 1 hour in blocking solution (5% fetal bovine serum, biosera, in 0,2% Triton/PBS). Sections were stained with a 1:750 dilution of rabbit primary antibody raised against CYP6CY3 in blocking solution for 12 to 16 hours at 4°C. Slides were washed using 0.2% Triton/PBS (3 × 15 min) followed by a 1-hour incubation in the dark using a 1:1000 dilution of goat anti-rabbit (Alexa Fluor 488, Molecular Probes) in blocking solution. Following this, slides were subjected to a single 5-min wash with 0.2% Triton/PBS and 2 × 5-min washes using 0.2% Tween/PBS. After ribonuclease A treatment (1:1000 dilution in 0.2% Tween/PBS), a 1:1000 dilution of To-PRO 3-Iodide in 0.2% Tween/PBS (Molecular Probes) was used to stain for nucleic acids. Last, slides were washed with 0.2% Tween/PBS before shielding them using one drop of VECTASHIELD mounting medium (Vector Laboratories). As controls, preimmune serums (in 1:750 dilutions) and anti-rabbit (Alexa Fluor 488, 1:1000) were tested in parallel with anti-IYS to check the specificity of the primary antibody. Imaging was conducted using a Leica SP8 laser-scanning microscope.

To examine the expression of CYP6CY3 in aphid guts, 50 guts from young aphids were dissected in 1× PBS and mechanically homogenized in 300 μl of lysis buffer [50 mM tris-HCl (pH 8.0), 150 mM NaCl, 0.1% SDS, 1% NP-40, 0.5% sodium deoxycholate, 5 mM EDTA (pH 8.0) and 5 mM EGTA (pH 8.0), 1 mM phenylmethylsulfonyl fluoride, and protease inhibitors; FASTTM Protease Inhibitor Cocktail, Sigma), followed by five cycles of freeze-thaw (liquid nitrogen/37°C in water bath) and centrifugation. Upon centrifugation of the lysate (95,000*g* for 2 hours at 4°C), the pellet that corresponded to microsomal proteins was dissolved in 5× Laemmli buffer, while the supernatant was acetone-precipitated and then resuspended in an equal volume of 1× Laemmli buffer. Last, the extracted polypeptides from both fractions, pellet and supernatant, were analyzed by SDS–polyacrylamide gel electrophoresis and Western blot. For this, the anti-CYP6CY3 was used at a 1:1000 dilution.

## Supplementary Material

aba1070_SM.pdf

aba1070_Data_file_S1.xlsx

aba1070_Data_file_S2.xlsx

## SUPPLEMENTARY MATERIALS

Supplementary material for this article is available at http://advances.sciencemag.org/cgi/content/full/6/19/eaba1070/DC1
