# Supplementary material for: The genetic architecture of a host shift: An adaptive walk protected an aphid and its endosymbiont from plant chemical defenses
Source: Sci Adv. 2020 May 6;6(19):eaba1070. doi: 10.1126/sciadv.aba1070 (PMC7202869; doi:10.1126/sciadv.aba1070)
Supplement: aba1070_SM.pdf [file aba1070_SM.pdf]

Science Advances
AAAS

advances.sciencemag.org/cgi/content/full/6/19/eaba1070/DC1

Supplementary Materials for

The genetic architecture of a host shift: An adaptive walk protected an aphid and its endosymbiont from plant chemical defenses

Kumar Saurabh Singh, Bartlomiej J. Troczka, Ana Duarte, Vasileia Balabanidou, Nasser Trissi, Leonela Z. Carabajal Paladino, Petr Nguyen, Christoph T. Zimmer, Kyriaki M. Papapostolou, Emma Randall, Bettina Lueke, Frantisek Marec, Emanuele Mazzoni, Martin S. Williamson, Alex Hayward, Ralf Nauen, John Vontas, Chris Bass*

*Corresponding author. Email: c.bass@exeter.ac.uk

Published 6 May 2020, *Sci. Adv.* **6**, eaba1070 (2020)
DOI: 10.1126/sciadv.aba1070

The PDF file includes:

Figs. S1 to S7
Tables S1 to S3
Legends for data files S1 and S2
References

Other Supplementary Material for this manuscript includes the following:

(available at advances.sciencemag.org/cgi/content/full/6/19/eaba1070/DC1)

Data files S1 and S2

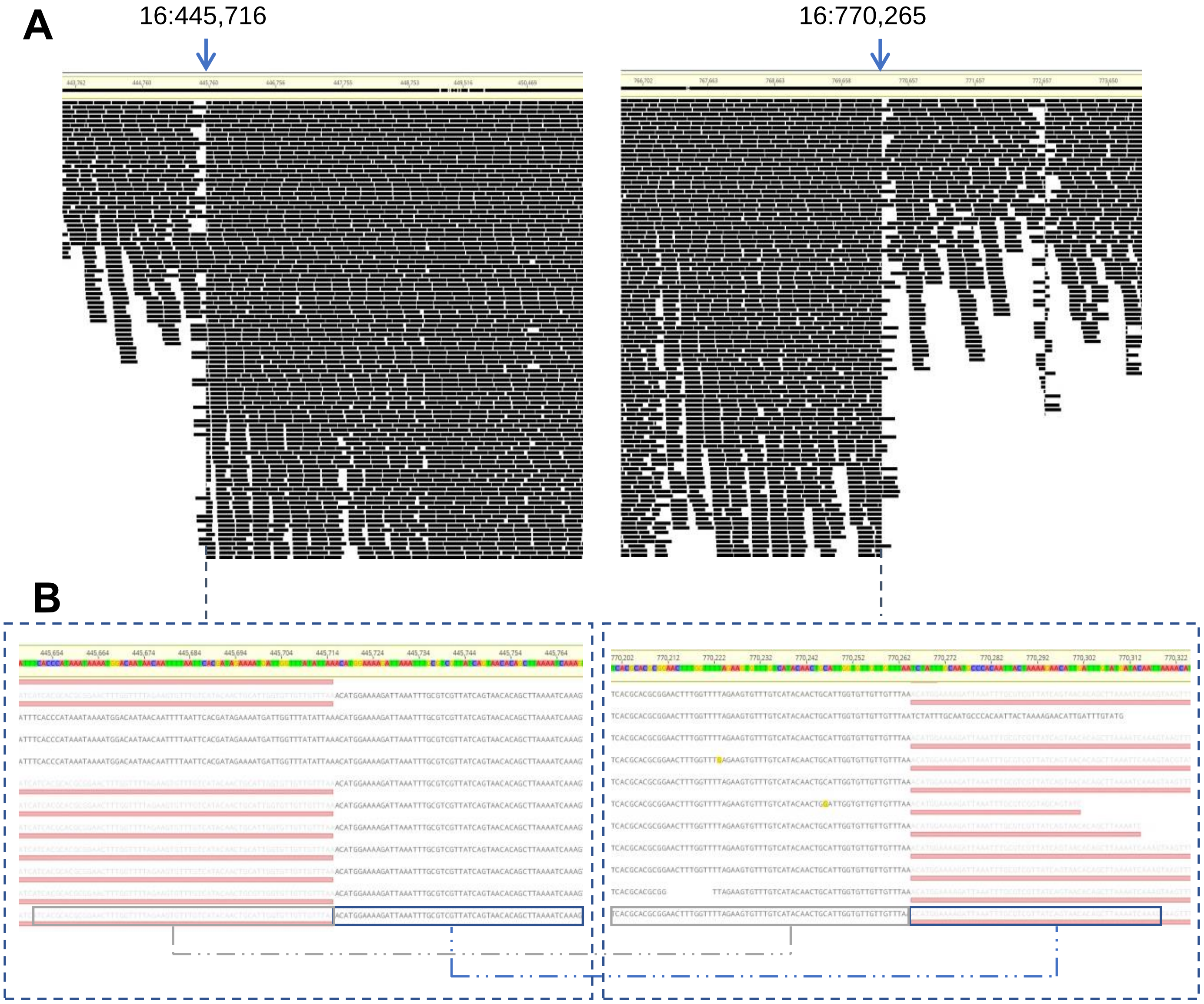

Fig. S1. DNAseq analysis reveals a large segmental duplication on scaffold 16 in *M. p. nicotianae*. DNAseq reads of *M. p. nicotianae* (reads from clone Mn1 are shown) mapped to scaffold 16 of the *M. persicae* reference genome, reveals evidence of genomic breakpoints at positions 16:445,716 and 16:770,265 in the form of a dramatic increase in coverage (**A**) and soft-clipped reads (**B**) that diverge in sequence after crossing these positions and link the two loci on individual reads.

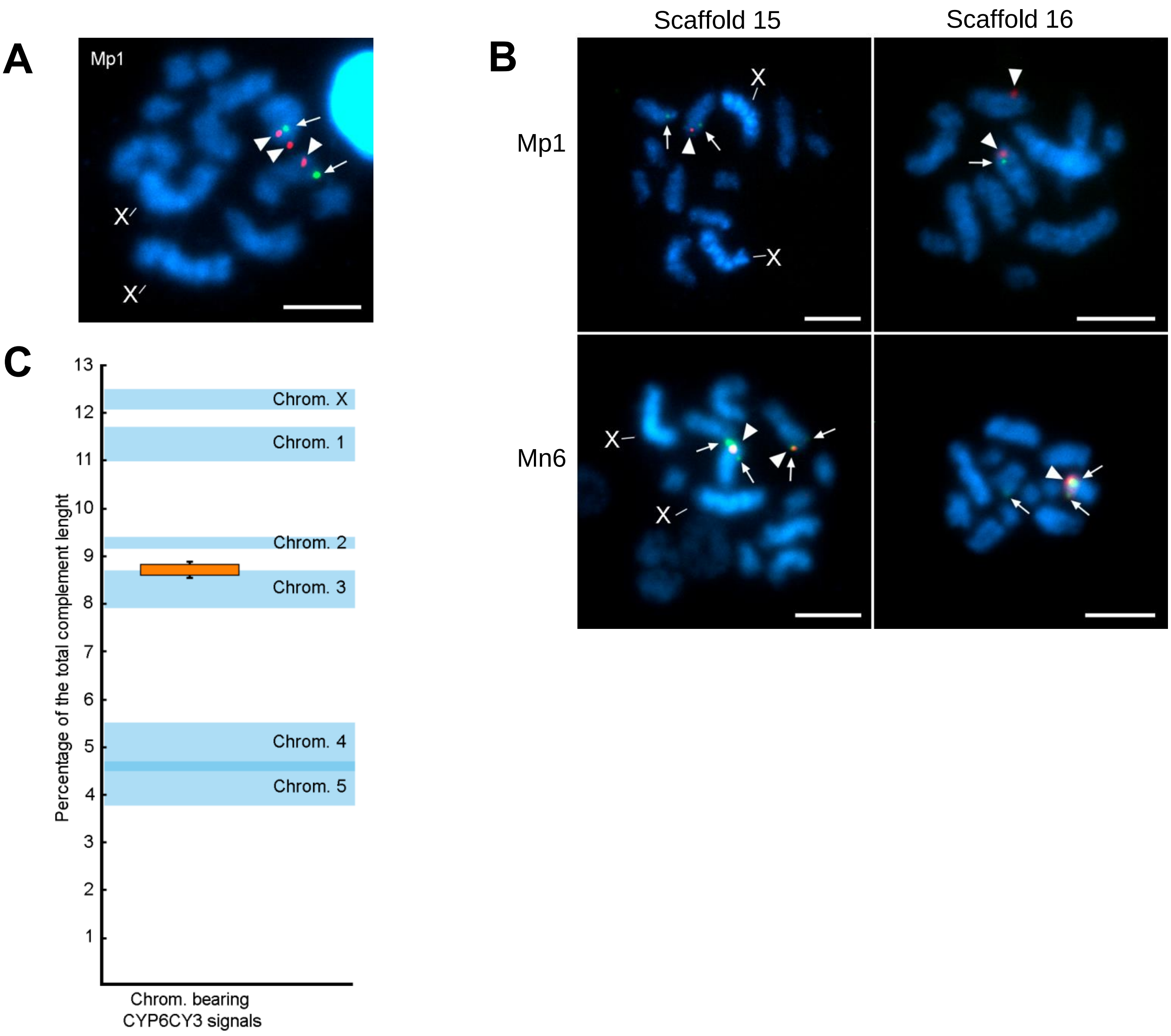

Fig. S2. Physical mapping of *CYP6CY3* on chromosomes of Mp1 and Mn6. (**A**) Co-localization of the BAC clone bearing a copy of *CYP6CY3* on scaffold 15 and a marker for scaffold 15 on metaphase chromosomes of Mp1 as revealed by combined BAC-FISH and TSA-FISH. The BAC was directly labelled with Cy3 (red, arrowheads), the scaffold 15 probe was detected by tyramide-FITC (green, arrows), and the chromosomes were counterstained with DAPI (blue). BAC-FISH was followed by TSA-FISH. The X chromosomes are identified by their size and heterochromatin content. Scale bar: 5 µm. (**B**) Co-localization of *CYP6CY3* and scaffolds 15 and 16 as revealed by double TSA-FISH on metaphase chromosomes of Mp1 and Mn6. The CYP6CY3 probe was detected by tyramide-Cy3 (red, arrowheads), the scaffold 15 and 16 probes were detected by tyramide-FITC (green, arrows), and the chromosomes were counterstained with DAPI (blue). Scale bar: 5 µm. (**C**) Identification of the chromosome pair bearing the CYP6CY3 signals in Mp1. The box-and-whisker plot (orange) represents the average percentage of the karyotype that corresponds to the chromosomes bearing the signals, with standard error and confidence interval of the mean. The percentage of the karyotype length corresponding to the six chromosome pairs of Mp1 (*18*) is shown in light blue.

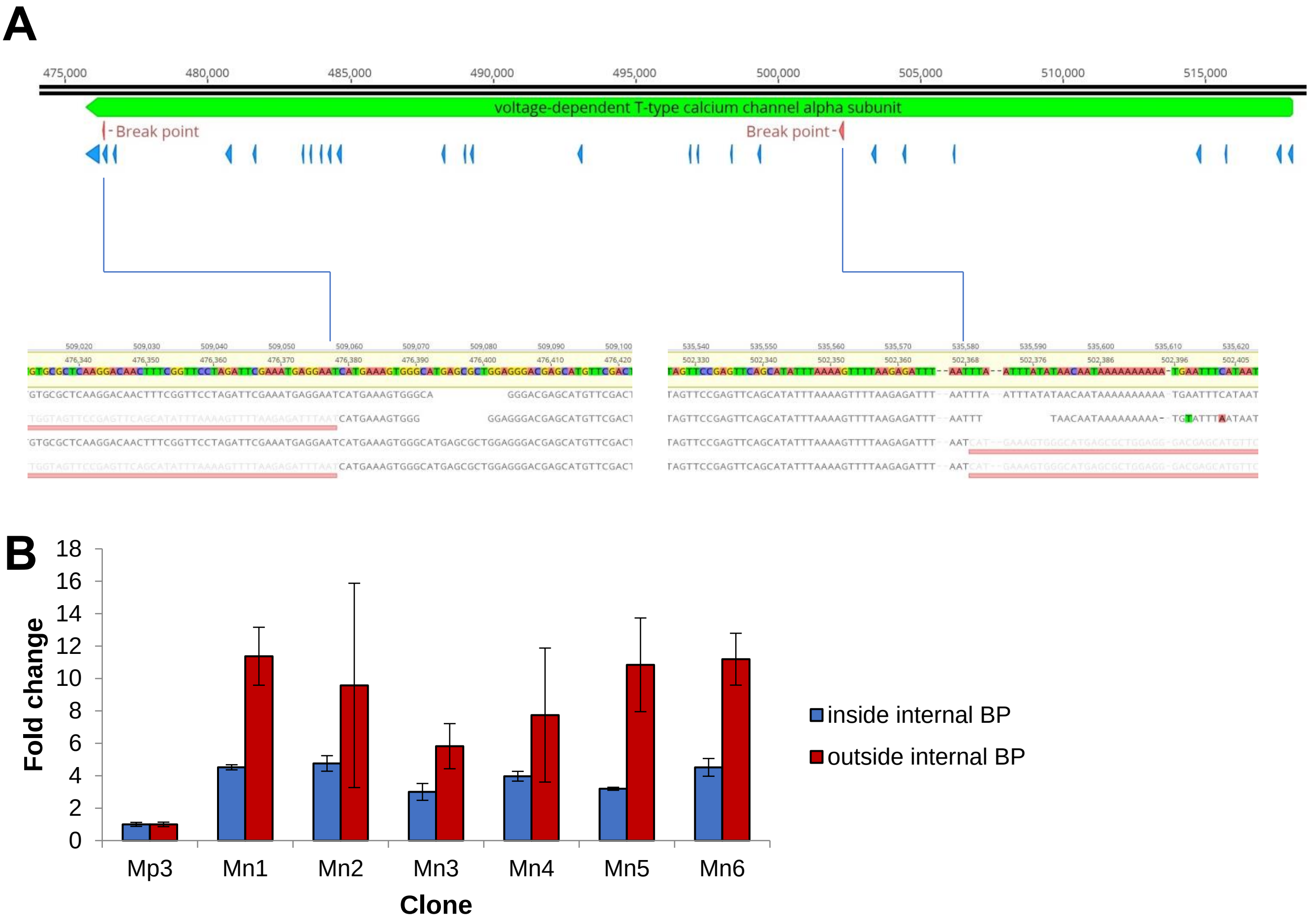

Fig. S3. Evidence for an internal duplication in gene copies of the T-type calcium channel. (**A**) Schematic of the T-type calcium channel gene (exons are shown as blue triangles) on scaffold 16. Break points are observed in exon 24 and intron 7 in the form of soft-clipped reads (annotated with pink bar) that link the sequences at these two positions on single reads, suggestive of an internal duplication. (**B**) Results of qPCR using primers within (red bars) and outside (blue bars) the predicted duplicated region (defined by break points, BP) confirming an increase in copy number within this region in all six *M. p. nicotianae clones* (Mn1-6) but not in *M. persicae* (Mp3). Error bars indicate 95% confidence limits (n=4).

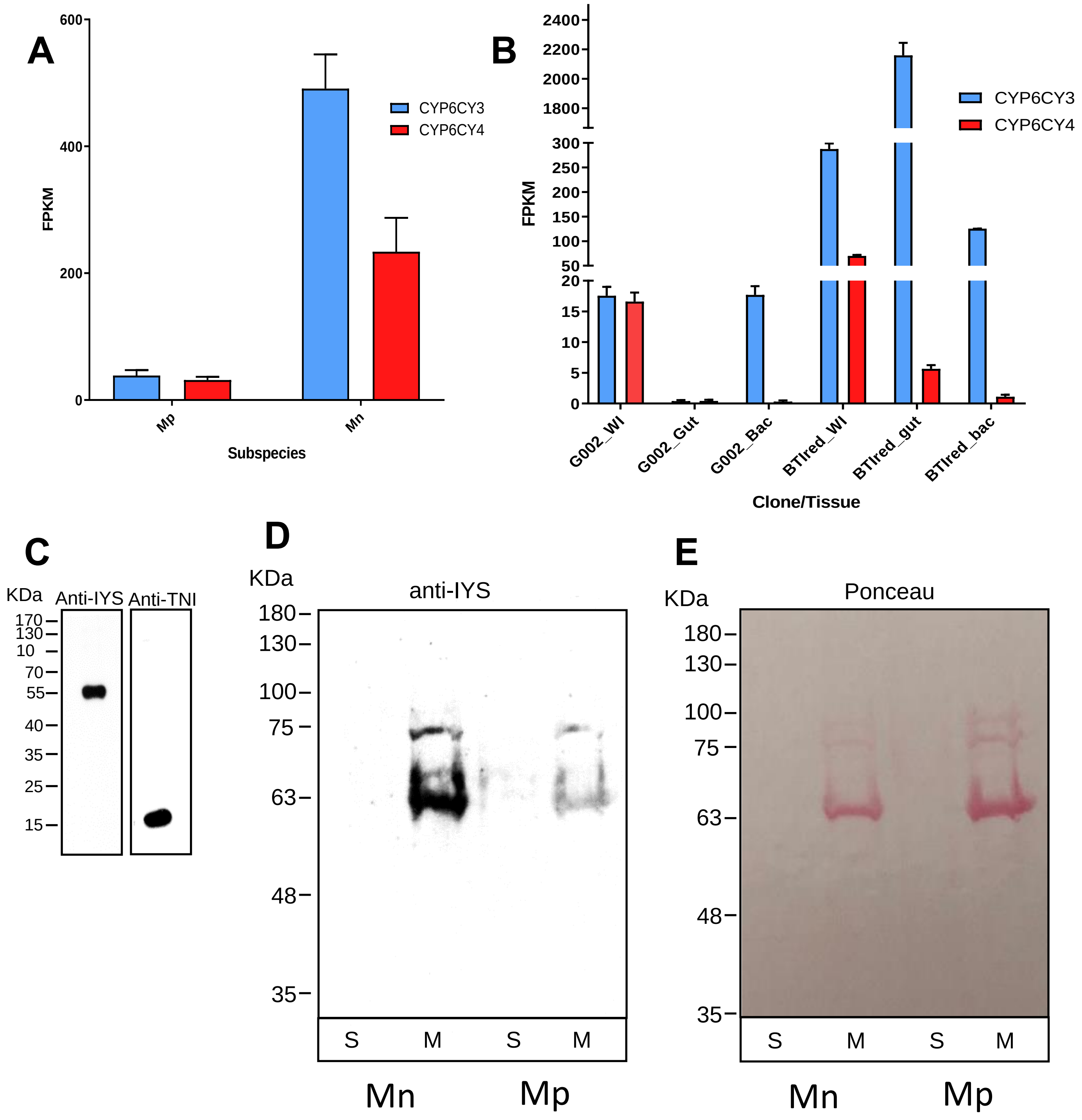

Fig. S4. Expression of *CYP6CY3* and *CYP6CY4* in *Myzus persicae* and *Myzus persicae nicotianae*. (**A**) Average expression of the P450 genes *CYP6CY3* and *CYP6CY4* in *M. persicae* (Mp) and *M. p. nicotianae* (Mn) derived from RNAseq data. Results are displayed as Fragments Per Kilobase of transcript per Million mapped reads (FPKM). Error bars indicate 95% CLs (n=6). (**B**) Expression of *CYP6CY3*, and *CYP6CY4*, in whole aphids (WI), gut and bacteriocyte (Bac) of the *M. persicae* clone G002 and *M. p. nicotianae* clone BTIred as determined by RNAseq analysis. Error bars indicate 95% confidence intervals of calculated FPKM values as generated in Cufflinks. (**C**) Reactivity of anti-CYP6CY3 peptide antibodies. Bacterial membranes expressing CYP6CY3 were loaded on a 10% SDS-PAGE gel and the separated polypeptides were transferred on nitrocellulose. Upon transfer, anti-IYS and anti-TNI in 1/1000 dilution were tested (the two antibodies were affinity purified against the corresponding peptides, IYS and TNI). (**D**, **E**) Expression of CYP6CY3 in guts of *M. persicae* and *M. p. nicotianae* as determined by Western blot. Soluble protein fraction (S) and microsomal protein fraction (M) extracted from 50 guts of *M. persicae* and *M. p. nicotianae*. (**D**) Nitrocellulose membrane labelled using anti-CYP6CY3 (anti-IYS). Same membrane stained with Ponceau (as a loading control) is shown in (**E**).

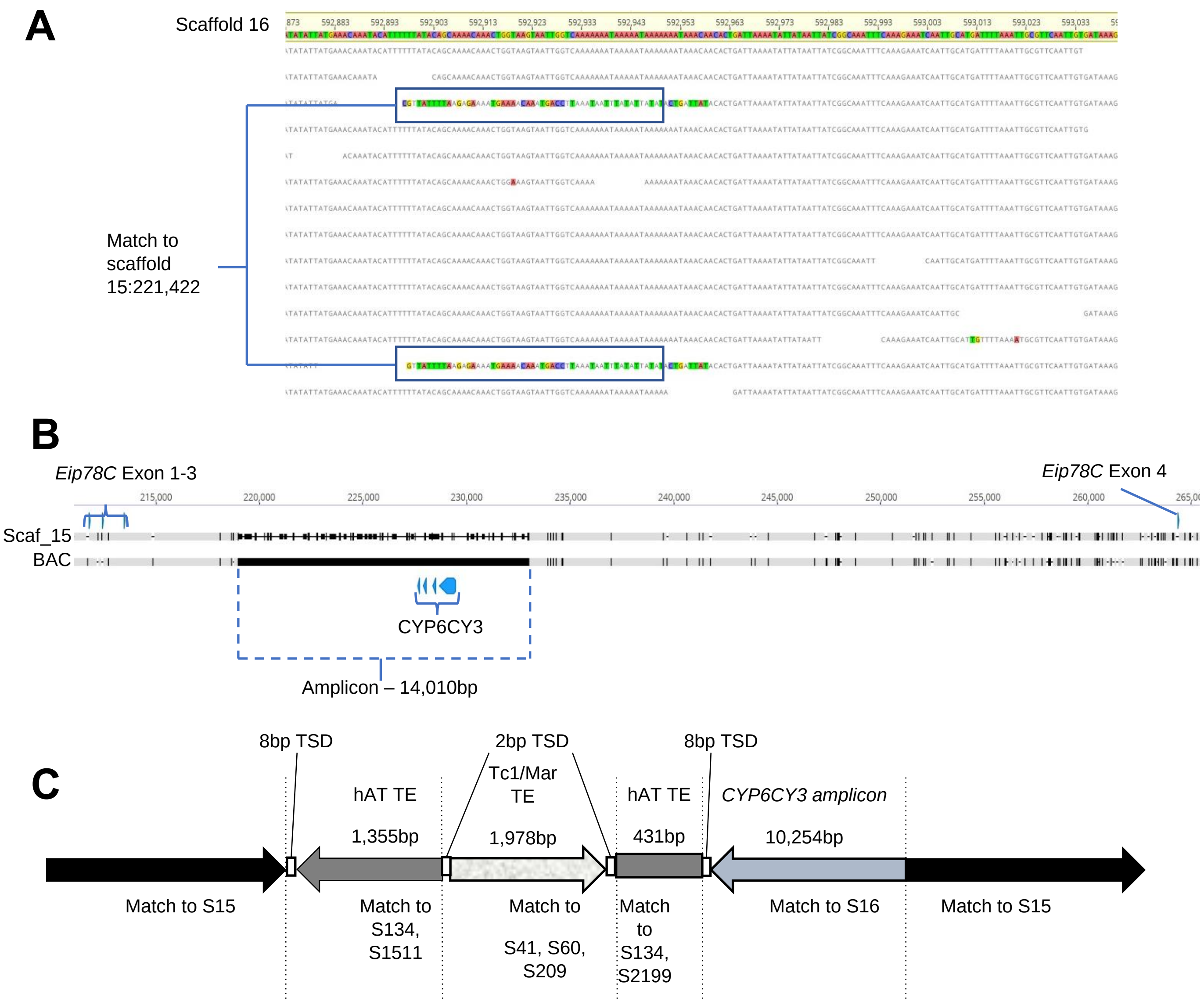

Fig. S5. An additional copy of the P450 *CYP6CY3* is observed on scaffold 15. (**A**) Mapping DNAseq data to scaffold 16 reveals evidence of an additional breakpoint at position 16:592,958 in all *M. p. nicotianae* clones (representative data is shown here and raw sequence data has been deposited in NCBI) in the form of divergent reads that link sequence at this position on scaffold 16 with sequence from scaffold 15 (15:221,422) on single reads. (**B**) Sequence alignment of the BAC assembly and scaffold 15 reveals that the additional copy of *CYP6CY3* has inserted into an intron of the *Eip78C* gene. (**C**) Schematic of the *CYP6CY3* amplicon showing regions of homology to scaffolds in the reference genome of *M. persicae.*

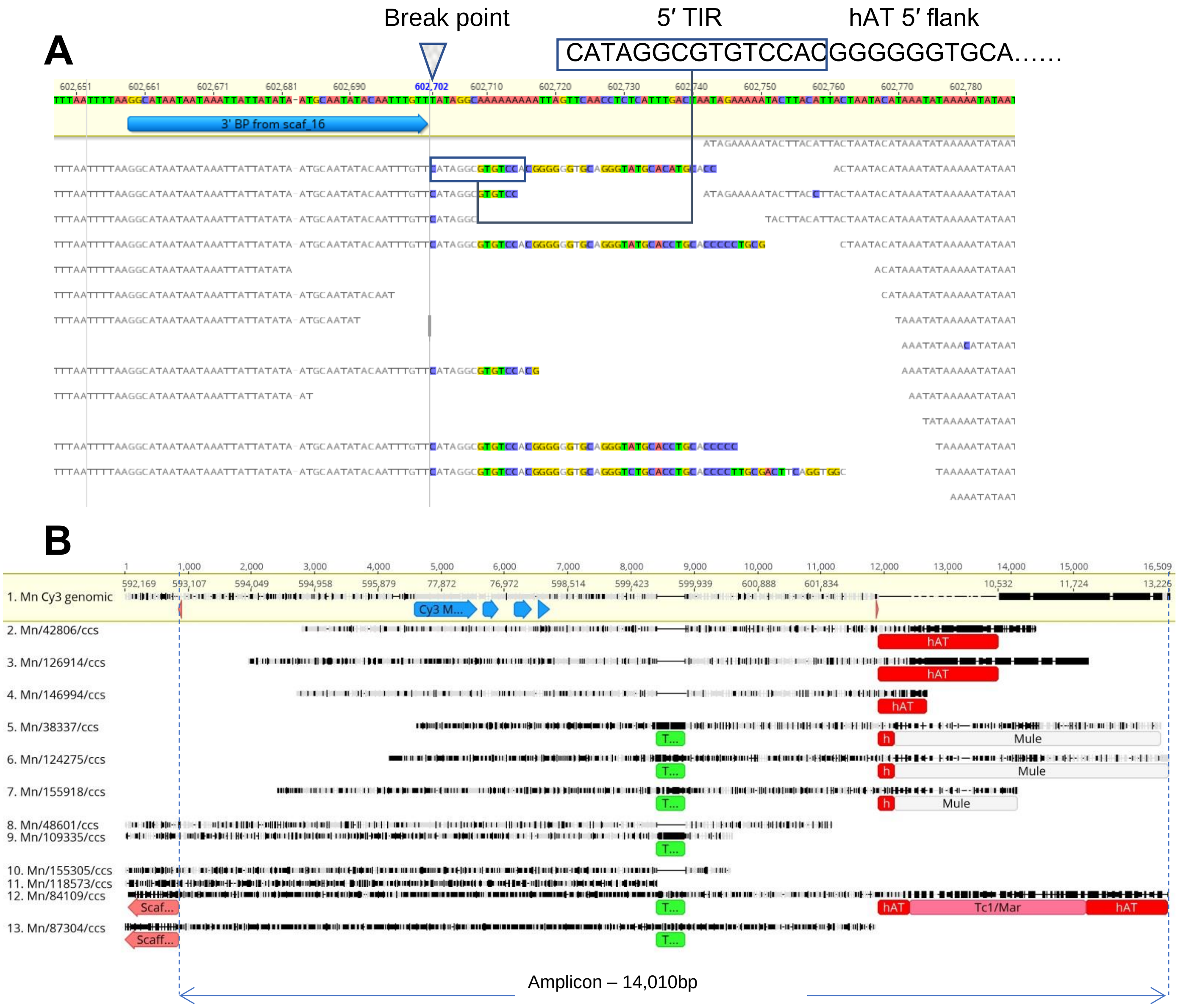

Fig. S6. Transposable element insertion events at the *CYP6CY3* locus in *Myzus p. nicotianae*. (**A**) All copies of *CYP6CY3* in the genome of *M. p. nicotianae* are associated with the hAT transposable element (hAT TE). DNAseq reads of all *M. p. nicotianae* clones were mapped to scaffold 16 of the *M. persicae s.s.* genome G006 at the site of the hAT insertion (identified by BAC sequencing), a representative example of mapped reads is shown. The sequence boxed corresponds to the terminal inverted repeat of the hAT TE. (**B**) Long PacBio circular consensus sequence reads obtained from the clone Mn1 aligned to scaffold 16 of the reference *M. persicae* G006 genome confirm this finding (the hAT TE is annotated in red) and show that two additional transposons (a TTAA3 element annotated in green and a Mule element annotated in grey) are associated with some but not all copies of *CYP6CY3* on scaffold 16. Finally *CYP6CY3* copies associated with scaffold 15 (bottom two reads) carry the TTAA3 transposon, and the hAT element into which the Tc1/Mariner element (annotated in pink) has inserted.

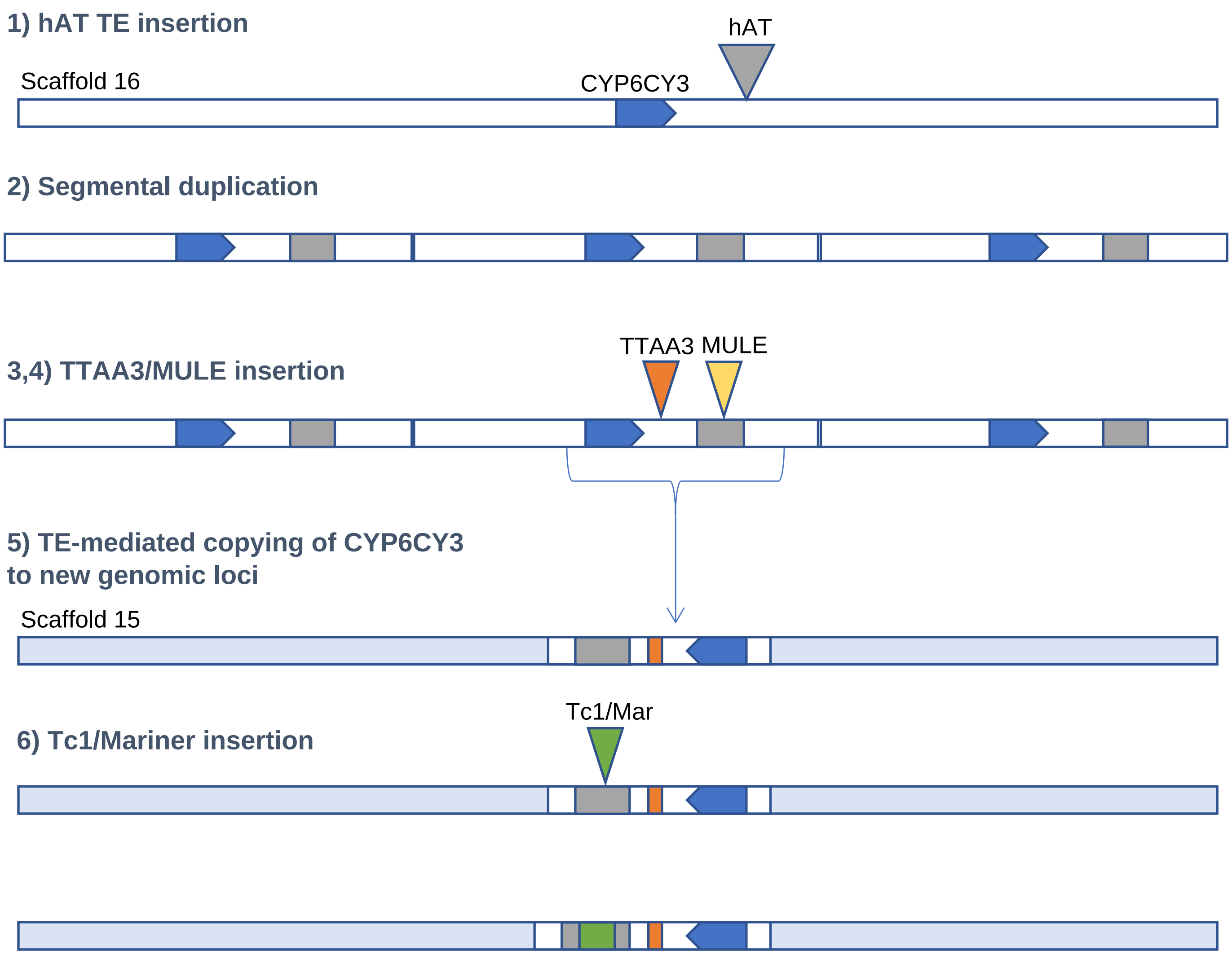

Fig. S7. Model of the mutational events leading to *CYP6CY3* amplification. 1) The hAT element inserted downstream of *CYP6CY3* on scaffold 16 prior to the segmental duplication (2). 3-4) Following segmental duplication the TTAA3 and Mule elements inserted in one of the copies of *CYP6CY3* on scaffold 16. 5) An amplicon incorporating the TTAA3 and hAT elements inserted into scaffold 15, a new loci on the same chromosome as scaffold 16, in reverse orientation. 6) A Tc1/Mariner element then inserted into the hAT element downstream of *CYP6CY3* on scaffold 15.

Table S1. Frequency of nonfunctionalizing mutations within the *SRC42A* and the T-type calcium channel genes in different clones of *Myzus persicae nicotianae*.

	Src42A 6bp deletion at position 16:454,566			Src42A 13bp insertion at position 16:455,092			T-type CaCh duplication at position 16:476,378		
Clone	reads with deletion	wildtype reads	frequency of reads with deletion	reads with insertion	wild type reads	frequency of reads with insertion	divergent reads	wild type reads	frequency of divergent reads
Mn1 DNA	not present	na	na	20	113	15.04%	92	112	45.09%
Mn1 RNA	na	na	na	21	311	6.33%	14	55	20.29%
Mn2 DNA	9	102	8.10%	18	112	13.80%	86	110	43.88%
Mn2 RNA	39	227	14.66%	24	377	5.99%	20	21	48.78%
Mn3 DNA	55	30	64.70%	not present	na	na	64	96	40%
Mn3 RNA	206	204	50.24%	na	na	na	13	21	38.24%
Mn4 DNA	10	114	8.06%	24	111	17.78%	94	100	48.45%
Mn4 RNA	48	345	12.21%	24	377	5.99%	24	49	32.88%
Mn5 DNA	46	69	40%	not present	na	na	71	88	44.65%
Mn5 RNA	176	169	51.01%	na	na	na	27	34	44.26%
Mn6 DNA	10	107	8.54%	48	95	33.57%	39	124	23.93%
Mn6 RNA	73	359	16.90%	102	546	15.70%	26	110	19.12%

Table S2. Sequences of the oligonucleotide primers used in this study.

Primer name	Sequence	Purpose
CYP6CY3-A_F	AGCCAAATCGGTTCTACTGT	FISH (Probe A)
CYP6CY3-A_R	TTTGTCGATGTTACAGTGCG	FISH (Probe A)
CYP6CY3-B_F	TGATAGGCACATCAATACAGTTAAC	FISH (Probe B)
CYP6CY3-B_R	CACAATTTTCTCAGGTTACGAACTGT	FISH (Probe B)
CYP6CY3-C_F	GATCAAAGAGTGTGGAGACG	FISH (Probe C)
CYP6CY3-C_R	TGAAACTCGAAGAGTCACCT	FISH (Probe C)
Scaffold 15_F	TGGAAGGCTTTATTCACACTCCA	FISH (Probe for scaffold 15)
Scaffold 15_R	ACTTAACTGTCTAATCTCGCCCA	FISH (Probe for scaffold 15)
Scaffold 16_F	GGTGCTTTACTAGTATACGACCTATGTT	FISH (Probe for scaffold 15)
Scaffold 16_R	CCACACATTTATGAGGTATTTGCTCG	FISH (Probe for scaffold 15)
Subtelomeric repeats_F	CGAATTTCGGGAATTTCAAAG	FISH (Probe for subtelomeric repeats)
Subtelomeric repeats_R	TTCAAAATTGTCTCACGACCTG	FISH (Probe for subtelomeric repeats)
CY3-15_F	CAC CAT TGA TAC AAG TCA TAC AA	Screening BAC library for CYP6CY3 on scaffold 15
CY3-15_R	GAT TCT TTA TCA CAA TTG AAC GC	Screening BAC library for CYP6CY3 on scaffold 15
q_c/gDNA_AD_nonamp_F2	GATGTCAATGGTTCCCGAGTTAC	QPCR of ADAMTS9 gene region outside amplicon
q_c/gDNA_AD_nonamp_R2	GTCACCGCCATAAAGAATATCGTG	QPCR of ADAMTS9 gene region outside amplicon
q_c/gDNA_AD_amp F2	CGTACGGTAAACTGGTGGAG	QPCR of ADAMTS9 gene region inside amplicon
q_c/gDNA_AD_amp R2	ATTCGGATACAATCACTGACGAC	QPCR of ADAMTS9 gene region inside amplicon
q_c/gDNA_Rib_nonamp F1	TGAAACCTTTCTTTGCTCCTGTTC	QPCR of RPS11 gene region outside amplicon
q_c/gDNA_Rib_nonamp R1	GATGTGGAAATTGGAGATATTGTGAC	QPCR of RPS11 gene region outside amplicon
q_gDNA_Rib_amp F1	GGTTAATAATGACGTCGAATTGGTC	QPCR of RPS11 gene region inside amplicon
q_gDNA_Rib_amp R1	GCAACCTACCGTGTTCTTGAAC	QPCR of RPS11 gene region inside amplicon
q_cDNA_Rib_amp F1	GCTGATCAGACAGAAAAAGCTTTC	QPCR of RPS11 gene region inside amplicon
q_cDNA_Rib_amp R1	TTTGAAACCGAGACCAACGCTG	QPCR of RPS11 gene region inside amplicon
Mp_004498_3F	CCGTTTACCTGACAACCTAATACTG	QPCR of CYP6CY3
Mp_004498_3R	AGGACGTTTATCTTTGTTTTCGGGT	QPCR of CYP6CY3
q_CY4_F3	TATCAAATACCCAACGATTCATTAACTA	QPCR of CYP6CY4
q_CY4_R3	CTTTTCCGGGTCTTTATAATACTTAGG	QPCR of CYP6CY4
q_CY23_F1	CCCTCCATTAGTTTTTTTGTTAAGAG	QPCR of CYP6CY23
q_CY23_R1	CTATCGTGATGTATTGCATACGAC	QPCR of CYP6CY23
hypo_prot_F1	AGAAATTCCGCATTTGTTTGCG	QPCR of gene encoding protein of unknown function
hypo_prot_R1	CGCCAGTGAATCCGATAAACC	QPCR of gene encoding protein of unknown function
q_c/gDNA_SRC42A_F1	CAAGAAGGACATGACGGGCTC	QPCR of SRC42A
q_c/gDNA_SRC42A_R1	CGGTCCATTCCTGACTTCACG	QPCR of SRC42A
q_c/gDNA_CaCh1_F1	TTTCTCTTGACGCTTGCCTCG	QPCR of t-type calcium channel outside the duplicated region of this gene
q_c/gDNA_CaCh1_R1	TCGACGTACCACTTTTGCGG	QPCR of t-type calcium channel outside the duplicated region of this gene
CaCh_dup_F3	GAAATAGAACACCTTGCGGACG	QPCR of t-type calcium channel inside the duplicated region of this gene
CaCh_dup_R3	CACGGTCAATTACGTTCTGGC	QPCR of t-type calcium channel

		inside the duplicated region of this gene
Actin_F	GGTGTCTCACACACAGTGCC	qPCR of reference gene (voltage-gated sodium channel)
Actin_R	CGGCGGTGGTGGTGAAGCTG	qPCR of reference gene (actin)
Para_F	GACCACGAGCTTCCCCGGTG	qPCR of reference gene (voltage-gated sodium channel)
Para_R	TGGTATACACGTTGGTTCTC	qPCR of reference gene (voltage-gated sodium channel)
GroEL F1	CTGGTGGTGGTGTTGCATTAG	qPCR of GroEL gene of *Buchnera aphidicola*
GroEL R1	CGCAAAGCAACTCGAATACCT	qPCR of GroEL gene of *Buchnera aphidicola*
RPS11/ADAMTS9_F1	TTT CTC TGT ACA TTA TTC AAC ATG	PCR validation of RPS11/ADAMTS9
RPS11/ADAMTS9_R1	GCA ACT ATT GAC ATT ACA CAT AG	PCR validation of RPS11/ADAMTS9
D099 pUAST F	TCACTGGAACTAGGCTAGCA	Sequence validation of transgenic flies
D102 pUAST F	GGATCCAAGCTTGCATGCCTG	Sequence validation of transgenic flies
D100 pUAST R	AAAGGCATTCCACCACTGCT	Sequence validation of transgenic flies
D101 pUAST R	CCACCACTGCTCCCATTCAT	Sequence validation of transgenic flies

Table S3. Details of methods used in Fluorescence In Situ Hybridization (FISH) experiments.

Experiment	Protocol (reference)	Probe labelling (dUTP-X / method / length of incubation at 15 °C / reference)	Probe cocktail (in 50% deionised formamide and 10% dextran sulfate in 2xSSC)	Modifications
A- Mapping of CYP6CY3	Simple TSA-FISH (Carabajal Paladino et al. 2014) (*31*)	dUTP-Digoxigenin / Nick translation kit / 1h 45m / Carabajal Paladino et al. 2014 (*31*)	40 ng CYP6CY3	Tyramide incubation: 10 min
B- Localization of CYP6CY3 and scaffolds 15 and 16	Double TSA-FISH (Carabajal Paladino et al. 2014) (*31*)	CYP6CY3: dUTP-Dinitrophenol / Nick translation / 1h 15m / Kato et al. 2006 *(35)*<br><br>Scaffold 15 or Scaffold 16: dUTP-Fluorescein / Nick translation kit / 2h / Carabajal Paladino et al. 2014 (*31*)	40 ng CYP6CY3<br>40 ng Scaffold 15 or Scaffold 16	Pre-treatments (steps 1-4 followed by three 5 min washes in 1xPBS):<br>1. 100µg/ul RNase in 2xSSC at 37°C for 1h<br>2. 50 µg/ml pepsin in 0.01 M HCl at 37°C for 5 min<br>3. 2% formaldehyde at room temperature (RT) for 10 min<br>4. 1% hydrogen peroxide in PBS at RT for 30 min<br>5. 5% Denhardt's solution at 37°C for 30 min<br>Denaturation of slides: 68°C for 3 min<br>Stringent washings:<br>1. 50% formamide in 2x SSC at 46°C, three times for 5 min each<br>2. 2xSSC at 46°C, three times for 5 min each<br>3. 0.1xSSC at 62°C, three times for 5 min each<br>Tyramide incubaton: 7 min
C- Localization of BAC and scaffolds 15 and 16	BAC-FISH (Yoshido et al. 2005) (*32*) followed by TSA-FISH re-probing (Carabajal Paladino et al. 2014) (*31*)	BAC: dUTP-Cy3 / Nick translation kit / 4h / Nguyen et al. 2013 (*34*)	250 ng BAC<br>2 ug competitor gDNA of clone Mp3	BAC-FISH slide pre-treatments: same as for Experiment B (Colocalization of CYP6CY3 and scaffolds 15 and 16)<br>Denaturation of slides: 68°C for 3 min
		Scaffold 15 or Scaffold 16: dUTP-Fluorescein / Nick translation kit / 2h / Carabajal Paladino et al. 2014 (*31*)	40 ng Scaffold 15 or Scaffold 16	Stripping steps:<br>1. 2xSSC at RT, three times for 5 mins each<br>2. 50% formamide 1% Triton in 0.1xSSC at 70°C, three times for 3 min each<br>Denaturation of slides: 70°C for 3 min<br>Stringent washings: same as for Experiment B (Colocalization of CYP6CY3 and scaffolds 15 and 16)<br>Denaturation of slides: 70°C for 3 min<br>Tyramide incubation: 7 min
	TSA-FISH (Carabajal Paladino et al. 2014) (*31*) followed by BAC-FISH (Yoshido et al. 2005) (*32*)	Scaffold 16: dUTP-Fluorescein / Nick translation kit / 2h / Nguyen et al. 2013 (*34*)	40 ng Scaffold 16	Pre-treatments: same as for Experiment B (Colocalization of CYP6CY3 and scaffolds 15 and 16)<br>Denaturation of slides: 68°C for 3 min<br>Tyramide incubaton: 7 min<br>After tyramide, denaturation of slides acc. to BAC-FISH protocol (Nguyen et al. 2013) and addition of BAC probe
		BAC: dUTP-Cy3 / Nick translation kit / 4h / Nguyen et al. 2013 (*34*)	250 ng BAC<br>2 ug competitor gDNA of clone Mp3	No slide pre-treatment

Data file S1. Lists of genes differentially expressed between 6 *Myzus persicae nicotianae* and 6 *Myzus persicae* s.s. clones. Each of the 36 comparisons is shown in a new tab.

Data files S2. Copy number variation (CNV) between 6 *Myzus persicae nicotianae* and 6 *Myzus persicae* s.s. clones (total of 36 comparisons) across scaffold 16. Regions with predicted CNV, the log fold values for each CNV region and associated confidence scores are shown. Each table corresponds to an individual comparison of sequence data from a *Myzus p. nicotianae* and *Myzus persicae s.s.* clone.

REFERENCES AND NOTES

1. M. Drès, J. Mallet, Host races in plant-feeding insects and their importance in sympatric speciation. *Philos. Trans. R. Soc. Lond. B Biol. Sci.* **357**, 471–492 (2002).

2. A. A. Forbes, S. N. Devine, A. C. Hippee, E. S. Tvedte, A. K. G. Ward, H. A. Widmayer, C. J. Wilson, Revisiting the particular role of host shifts in initiating insect speciation. *Evolution* **71**, 1126–1137 (2017).

3. K. W. Matsubayashi, I. Ohshima, P. Nosil, Ecological speciation in phytophagous insects. *Entomol. Exp. Appl.* **134**, 1–27 (2009).

4. J. S. Bernal, R. F. Medina, Agriculture sows pests: How crop domestication, host shifts, and agricultural intensification can create insect pests from herbivores. *Curr. Opin. Insect Sci.* **26**, 76–81 (2018).

5. K. L. Vertacnik, C. R. Linnen, Evolutionary genetics of host shifts in herbivorous insects: Insights from the age of genomics. *Ann. N. Y. Acad. Sci.* **1389**, 186–212 (2017).

6. C. Bass, C. T. Zimmer, J. M. Riveron, C. S. Wilding, C. S. Wondji, M. Kaussmann, L. M. Field, M. S. Williamson, R. Nauen, Gene amplification and microsatellite polymorphism underlie a recent insect host shift. *Proc. Natl. Acad. Sci. U.S.A.* **110**, 19460–19465, (2013).

7. R. L. Blackman, Morphological discrimination of a tobacco-feeding form from *Myzus persicae* (Sulzer) (Hemiptera: Aphididae), and a key to New World *Myzus* (*Nectarosiphon*) species. *Bull. Entomol. Res.* **77**, 713–730 (1987).

8. V. Katju, U. Bergthorsson, Copy-number changes in evolution: Rates, fitness effects and adaptive significance. *Front. Genet.* **4**, 273 (2013).

9. R. L. Rogers, T. Bedford, D. L. Hartl, Formation and longevity of chimeric and duplicate genes in *Drosophila melanogaster*. *Genetics* **181**, 313–322 (2009).

10. R. Kelwick, I. Desanlis, G. N. Wheeler, D. R. Edwards, The ADAMTS (A Disintegrin and Metalloproteinase with Thrombospondin motifs) family. *Genome Biol.* **16**, 113 (2015).

11. S. Shigenobu, H. Watanabe, M. Hattori, Y. Sakaki, H. Ishikawa, Genome sequence of the endocellular bacterial symbiont of aphids *Buchnera* sp. APS. *Nature* **407**, 81–86 (2000).

12. L. Ding, J. Chen, J. Zou, L. Zhang, Y. Ye, Dynamic metabolomic responses of *Escherichia coli* to nicotine stress. *Can. J. Microbiol.* **60**, 547–556 (2014).

13. C. S. Pavia, A. Pierre, J. Nowakowski, Antimicrobial activity of nicotine against a spectrum of bacterial and fungal pathogens. *J. Med. Microbiol.* **49**, 674–675 (2000).

14. H. A. Orr, The genetic theory of adaptation: A brief history. *Nat. Rev. Genet.* **6**, 119–127 (2005).

15. E. B. Chuong, N. C. Elde, C. Feschotte, Regulatory activities of transposable elements: From conflicts to benefits. *Nat. Rev. Genet.* **18**, 71–86 (2016).

16. C. Feschotte, Transposable elements and the evolution of regulatory networks. *Nat. Rev. Genet.* **9**, 397–405 (2008).

17. J.-C. Simon, E. d'Alençon, E. Guy, E. Jacquin-Joly, J. Jaquiéry, P. Nouhaud, J. Peccoud, A. Sugio, R. Streiff, Genomics of adaptation to host-plants in herbivorous insects. *Brief. Funct. Genomics* **14**, 413–423, (2015).

18. V. Monti, G. Lombardo, H. D. Loxdale, G. C. Manicardi, M. Mandrioli, Continuous occurrence of intra-individual chromosome rearrangements in the peach potato aphid, *Myzus persicae* (Sulzer) (Hemiptera: Aphididae). *Genetica* **140**, 93–103 (2012).

19. T. E. Mittler, H. Kunkel, Wing production by grouped and isolated apterae of the aphid *Myzus persicae* on artificial diet. *Entomol. Exp. Appl.* **14**, 83–92 (1971).

20. S. Andrews, FastQC: a quality control tool for high throughput sequence data (2010); www.bioinformatics.babraham.ac.uk/projects/fastqc.

21. www.bioinformatics.babraham.ac.uk/projects/trim_galore/.

22. C. Trapnell, A. Roberts, L. Goff, G. Pertea, D. Kim, D. R. Kelley, H. Pimentel, S. L. Salzberg, J. L. Rinn, L. Pachter, Differential gene and transcript expression analysis of RNA-seq experiments with TopHat and Cufflinks. *Nat. Protoc.* **7**, 562–578 (2012).

23. H. Li, Aligning sequence reads, clone sequences and assembly contigs with BWA-MEM. arXiv.1303.3997v1 **[q-bio.GN].** (16 March 2013).

24. C. Xie, M. T. Tammi, CNV-seq, a new method to detect copy number variation using high-throughput sequencing. *BMC Bioinformatics* **10**, 80 (2009).

25. M. W. Pfaffl, A new mathematical model for relative quantification in real-time RT-PCR. *Nucleic Acids Res.* **29**, e45 (2001).

26. J. Vandesompele, K. De Preter, F. Pattyn, B. Poppe, N. V. Roy, A. De Paepe, F. Speleman, Accurate normalization of real-time quantitative RT-PCR data by geometric averaging of multiple internal control genes. *Genome Biol.* **3**, research0034.1 (2002).

27. J. B. Schenkman, I. Jansson, Spectral Analyses of Cytochromes P450. *In Cytochrome P450 Protocols*, I.R. Phillips, E. A. Shephard, Eds. (Humana Press, 2006).

28. F. Zhu, R. Parthasarathy, H. Bai, K. Woithe, M. Kaussmann, R. Nauen, D. A. Harrison, S. R. Palli, A brain-specific cytochrome P450 responsible for the majority of deltamethrin resistance in the QTC279 strain of *Tribolium castaneum*. *Proc. Natl. Acad. Sci. U.S.A.* **107**, 8557–8562 (2010).

29. C. Chen-Shan, D. H. Alexander, P. Marks, A. A. Klammer, J. Drake, C. Heiner, A. Clum, A. Copeland, J. Huddleston, E. E. Eichler, S. W. Turner, J. Korlach, Nonhybrid, finished microbial genome assemblies from long-read SMRT sequencing data. *Nat. Methods* **10**, 563–569 (2013).

30. W. Traut, Pachytene mapping in the female silkworm *Bombyx mori* L. (Lepidoptera). *Chromosoma* **58**, 275–284 (1976).

31. L. Z. Carabajal Paladino, P. Nguyen, J. Šíchová, F. Marec, Mapping of single-copy genes by TSA-FISH in the codling moth, *Cydia pomonella*. *BMC Genet.* **15**, S15 (2014).

32. A. Yoshido, H. Bando, Y. Yasukochi, K. Sahara, The *Bombyx mori* Karyotype and the Assignment of Linkage Groups. *Genetics* **170**, 675–685 (2005).

33. A. Reeves, MicroMeasure: A new computer program for the collection and analysis of cytogenetic data. *Genome* **44**, 439–443 (2001).

34. P. Nguyen, M. Sýkorová, J. Šíchová, V. Kůta, M. Dalíková, R. Čapková Frydrychová, L. G. Neven, K. Sahara, F. Marec, Neo-sex chromosomes and adaptive potential in tortricid pests. *Proc. Natl. Acad. Sci. U.S.A.* **110**, 6931–6936 (2013).

35. A. Kato, P. S. Albert, J. M. Vega, J. A. Birchler, Sensitive fluorescence *in situ* hybridization signal detection in maize using directly labeled probes produced by high concentration DNA polymerase nick translation. *Biotech. Histochem.* **81**, 71–78 (2006).
